# Surgical interventions for Ménière’s disease

**DOI:** 10.1002/14651858.CD015249.pub2

**Published:** 2023-02-24

**Authors:** Ambrose Lee, Katie E Webster, Ben George, Natasha A Harrington-Benton, Owen Judd, Diego Kaski, Otto R Maarsingh, Samuel MacKeith, Jaydip Ray, Vincent A Van Vugt, Martin J Burton

**Affiliations:** Department of Otolaryngology - Head and Neck SurgeryUniversity of TorontoTorontoCanada; Cochrane ENT, Nuffield Department of Surgical SciencesUniversity of OxfordOxfordUK; Corpus Christi CollegeUniversity of OxfordOxfordUK; Ménière’s SocietyWootonUK; ENT DepartmentUniversity Hospitals of Derby and Burton NHS Foundation TrustDerbyUK; National Hospital for Neurology and NeurosurgeryLondonUK; Department of General Practice, Amsterdam UMCVrije Universiteit Amsterdam, Amsterdam Public Health Research InstituteAmsterdamNetherlands; ENT DepartmentOxford University Hospitals NHS Foundation TrustOxfordUK; University of SheffieldSheffieldUK; Cochrane UKOxfordUK

## Abstract

**Background:**

Ménière's disease is a condition that causes recurrent episodes of vertigo, associated with hearing loss and tinnitus. First‐line treatments often involve dietary or lifestyle changes, medication or local (intratympanic) treatments. However, surgery may also be considered for people with persistent or severe symptoms. The efficacy of different surgical interventions at preventing vertigo attacks, and their associated symptoms, is currently unclear.

**Objectives:**

To evaluate the benefits and harms of surgical interventions versus placebo or no treatment in people with Ménière's disease.

**Search methods:**

The Cochrane ENT Information Specialist searched the Cochrane ENT Register; Central Register of Controlled Trials (CENTRAL); Ovid MEDLINE; Ovid Embase; Web of Science; ClinicalTrials.gov; ICTRP and additional sources for published and unpublished trials. The date of the search was 14 September 2022.

**Selection criteria:**

We included randomised controlled trials (RCTs) and quasi‐RCTs in adults with definite or probable Ménière's disease comparing ventilation tubes, endolymphatic sac surgery, semi‐circular canal plugging/obliteration, vestibular nerve section or labyrinthectomy with either placebo (sham surgery) or no treatment. We excluded studies with follow‐up of less than three months, or with a cross‐over design (unless data from the first phase of the study could be identified).

**Data collection and analysis:**

We used standard Cochrane methods. Our primary outcomes were: 1) improvement in vertigo (assessed as a dichotomous outcome ‐ improved or not improved), 2) change in vertigo (assessed as a continuous outcome, with a score on a numerical scale) and 3) serious adverse events. Our secondary outcomes were: 4) disease‐specific health‐related quality of life, 5) change in hearing, 6) change in tinnitus and 7) other adverse effects. We considered outcomes reported at three time points: 3 to < 6 months, 6 to ≤ 12 months and > 12 months. We used GRADE to assess the certainty of evidence for each outcome.

**Main results:**

We included two studies with a total of 178 participants. One evaluated ventilation tubes compared to no treatment, the other evaluated endolymphatic sac decompression compared to sham surgery.

**Ventilation tubes**

We included a single RCT of 148 participants with definite Ménière's disease. It was conducted in a single centre in Japan from 2010 to 2013. Participants either received ventilation tubes with standard medical treatment, or standard medical treatment alone, and were followed up for two years. Some data were reported on the number of participants in whom vertigo resolved, and the effect of the intervention on hearing. Our other primary and secondary outcomes were not reported in this study. This is a single, small study and for all outcomes the certainty of evidence was low or very low. We are unable to draw meaningful conclusions from the numerical results.

**Endolymphatic sac decompression**

We also included one RCT of 30 participants that compared endolymphatic sac decompression with sham surgery. This was a single‐centre study conducted in Denmark during the 1980s. Follow‐up was predominantly conducted at one year, but additional follow‐up continued for up to nine years in some participants. Some data were reported on hearing and vertigo (both improvement in vertigo and change in vertigo), but our other outcomes of interest were not reported. Again, this is a single, very small study and we rated the certainty of the evidence as very low for all outcomes. We are therefore unable to draw meaningful conclusions from the numerical results.

**Authors' conclusions:**

We are unable to draw clear conclusions about the efficacy of these surgical interventions for Ménière's disease. We identified evidence for only two of our five proposed comparisons, and we assessed all the evidence as low‐ or very low‐certainty. This means that we have very low confidence that the effects reported are accurate estimates of the true effect of these interventions. Many of the outcomes that we planned to assess were not reported by the studies, such as the impact on quality of life, and adverse effects of the interventions. Consensus on the appropriate outcomes to measure in studies of Ménière's disease is needed (i.e. a core outcome set) in order to guide future studies in this area and enable meta‐analyses of the results. This must include appropriate consideration of the potential harms of treatment, as well as the benefits.

## Plain language summary

**What are the benefits and risks of different types of surgery for Ménière's disease?**

**Key messages**

Due to a lack of robust evidence, it is not clear whether any types of surgery used to treat Ménière's disease work to improve people's symptoms. There was no information regarding the potential for serious risks from these treatments.

Larger, well‐conducted studies are needed in order to identify whether surgery may be effective, and assess whether there are any harmful effects of treatment.

Further work also needs to be done to find out how best to measure the symptoms of people with Ménière's disease, in order to assess whether treatments are beneficial or not. This should include the development of a 'core outcome set' ‐ a list of things that should be measured in all studies on Ménière's disease.

**What is Ménière's disease?**

Ménière's disease is a condition that affects the inner ear. It causes repeated attacks of dizziness or vertigo (a spinning sensation), together with hearing problems, tinnitus (ringing, humming or buzzing noises in the ears) and a feeling of fullness or pressure in the ear. It usually affects adults, and starts in middle age. Although it is a relatively uncommon condition, it can have a large impact on quality of life.

**How is Ménière's disease treated?**

There is a lot of variation in how Ménière's disease is treated in different places (both nationally and internationally). Currently there is no agreement on the best treatment strategy. Lifestyle and dietary advice and oral medications are often used as the initial treatment for Ménière's disease. Sometimes other treatments ‐ such as injections into the ear ‐ may also be used. If these treatments do not work, or if people have very severe symptoms, then surgery may be considered.

**What did we want to find out?**

We wanted to find out:

‐ whether there was evidence that any types of surgery work to reduce the symptoms of Ménière's disease;

‐ whether the treatments might cause any harm.

**What did we do?**

We searched for studies that compared different types of surgery to either no treatment or sham surgery.

**What did we find?**

We found two studies, which included a total of 178 people.

*Ventilation tubes*

The first study evaluated ventilation tubes (sometimes known as grommets) ‐ small tubes that are inserted into the eardrum. It was unclear whether this treatment made a difference to vertigo symptoms, and the study did not report on potential harms of the surgery.

*Endolymphatic sac decompression*

The second study looked at a specific type of surgery on the 'endolymphatic sac' ‐ a fluid filled part of the inner ear. The surgery involved placing a small tube to help drain fluid from this sac. People in this study either received this surgery, or a type of sham surgery, which should not have affected their symptoms of Ménière's disease at all. Again, it was unclear whether this treatment made a difference to vertigo symptoms, and the study did not report on potential harms of the surgery.

**What are the limitations of the evidence?**

We have very little confidence in the evidence because the two studies conducted were small and had problems in their conduct, which means that the results may be unreliable. Larger, well‐conducted studies are needed to try and work out how effective the different treatments really are.

**How up‐to‐date is this evidence?**

This evidence is up‐to‐date to September 2022.

## Summary of findings

**Summary of findings 1:** Ventilation tubes compared to no treatment for Ménière’s disease

**Ventilation tubes compared to no treatment for Ménière’s disease**						
**Patient or population:** Ménière’s disease **Setting:** outpatient **Intervention:** ventilation tubes **Comparison:** no treatment						
**Outcomes**	Anticipated absolute effects^*^ (95% CI)		**Relative effect (95% CI)**	**№ of participants (studies)**	**Certainty of the evidence (GRADE)**	**Comments**
	**Risk with no treatment**	**Risk with ventilation tubes**				
Improvement in frequency of vertigo (complete resolution) at > 12 months	Study population		RR 1.55 (1.22 to 1.97)	133 (1 RCT)	⊕⊝⊝⊝ Very low^1,2,3,4^	The evidence is very uncertain about the effect of ventilation tubes on improvement in vertigo at > 12 months.
	543 per 1000	841 per 1000 (662 to 1000)				
Change in frequency of vertigo	No study reported this outcome					
Serious adverse events	No study reported this outcome					
*The risk in the intervention group (and its 95% confidence interval) is based on the assumed risk in the comparison group and the **relative effect** of the intervention (and its 95% CI). **CI:** confidence interval; **RCT:** randomised controlled trial; **RR:** risk ratio						
**GRADE Working Group grades of evidence** **High certainty:** we are very confident that the true effect lies close to that of the estimate of the effect. **Moderate certainty:** we are moderately confident in the effect estimate: the true effect is likely to be close to the estimate of the effect, but there is a possibility that it is substantially different. **Low certainty:** our confidence in the effect estimate is limited: the true effect may be substantially different from the estimate of the effect. **Very low certainty:** we have very little confidence in the effect estimate: the true effect is likely to be substantially different from the estimate of effect.						

^1^Unblinded study. ^2^Risk of selective reporting for this outcome. ^3^Outcome considers 'complete resolution' of vertigo only, not 'any improvement'. ^4^Optimal information size was not reached, taken as > 300 events for dichotomous outcomes, as a rule of thumb.

**Summary of findings 2:** Endolymphatic sac decompression compared to placebo (sham surgery) for Ménière’s disease

**Endolymphatic sac decompression compared to placebo (sham surgery) for Ménière’s disease**						
**Patient or population:** adults with Ménière’s disease **Setting:** outpatients **Intervention:** endolymphatic sac decompression **Comparison:** sham surgery						
**Outcomes**	Anticipated absolute effects^*^ (95% CI)		**Relative effect (95% CI)**	**№ of participants (studies)**	**Certainty of the evidence (GRADE)**	**Comments**
	**Risk with sham surgery**	**Risk with endolymphatic sac decompression**				
Improvement in vertigo (complete resolution) at 6 to ≤ 12 months (12 months)	Study population		RR 1.30 (0.86 to 1.96)	30 (1 RCT)	⊕⊝⊝⊝ Very low^1,2,3,4^	The evidence is very uncertain about the effect of endolymphatic sac decompression on improvement (or resolution) in vertigo at 12 months.
	667 participants per 1000 would report that their vertigo had resolved	867 participants per 1000 would report that their vertigo had resolved (from 573 to 1000)				
Change in vertigo at 6 to ≤ 12 months (12 months) Assessed with: daily rating scale Scale from: 0 to 93, higher scores = worse symptoms	The mean change in vertigo was ‐10.4 points	MD 12.39 points lower (22.81 lower to 1.97 lower)	—	29 (1 RCT)	⊕⊝⊝⊝ Very low^1,3,4,5^	The evidence is very uncertain about the effect of endolymphatic sac decompression on the change in vertigo at 12 months, using a daily rating score.
Serious adverse events	No study reported this outcome					
*The risk in the intervention group (and its 95% confidence interval) is based on the assumed risk in the comparison group and the **relative effect** of the intervention (and its 95% CI). **CI:** confidence interval; **RCT:** randomised controlled trial; **RR:** risk ratio						
**GRADE Working Group grades of evidence** **High certainty:** we are very confident that the true effect lies close to that of the estimate of the effect. **Moderate certainty:** we are moderately confident in the effect estimate: the true effect is likely to be close to the estimate of the effect, but there is a possibility that it is substantially different. **Low certainty:** our confidence in the effect estimate is limited: the true effect may be substantially different from the estimate of the effect. **Very low certainty:** we have very little confidence in the effect estimate: the true effect is likely to be substantially different from the estimate of effect.						

^1^Multiple domains were rated at unclear risk of bias. Re‐analysis of the original trial data by Welling 2000 indicates that the analysis used may have been inappropriate. ^2^Outcome assessed is 'complete resolution' of vertigo, not any improvement. ^3^The criteria used for the diagnosis of Ménière's disease were poorly defined, therefore the population may not be appropriate. ^4^Sample size is extremely small. Fails to meet optimal information size and confidence intervals are very wide. ^5^An unvalidated scale was used to assess vertigo.

## Background

### Description of the condition

Ménière's disease was first described by Prosper Ménière in 1861 as a condition characterised by episodes of vertigo, associated with hearing loss and tinnitus (Baloh 2001). Sufferers may also report a feeling of fullness in the affected ear. Typically, it initially affects one ear, although some individuals may progress to develop bilateral disease. A hallmark of the condition is that symptoms are intermittent ‐ occurring as discrete attacks that last from minutes to several hours, then resolve. However, over time there is usually a gradual deterioration in hearing, and there may be progressive loss of balance function, leading to chronic dizziness or vertigo.

The diagnosis of Ménière's disease is challenging, due to the episodic nature of the condition, clinical heterogeneity and the lack of a 'gold standard' diagnostic test. Even the agreed, international classification system has scope for two categories of diagnosis – 'definite' and 'probable' (Lopez‐Escamez 2015). In brief, a diagnosis of definite Ménière's disease requires at least two episodes of vertigo, each lasting 20 minutes to 12 hours, together with audiometrically confirmed hearing loss and fluctuating aural symptoms (reduction in hearing, tinnitus or fullness) in the affected ear. 'Probable' Ménière's disease includes similar features, but without the requirement for audiometry to diagnose hearing loss, and with scope for the vertigo episodes to last longer (up to 24 hours). Both categories ('definite' and 'probable') require that the symptoms are not more likely to be due to an alternative diagnosis, due to the recognised challenges in distinguishing between balance disorders.

Given the difficulties in diagnosis, the true incidence and prevalence of the disease are difficult to ascertain. A population‐based study in the UK using general practice data estimated the incidence to be 13.1 per 100,000 person‐years (Bruderer 2017), and the prevalence of the disease has been estimated at 190 per 100,000 people in the US (Harris 2010). It is a disorder of mid‐life, with diagnosis typically occurring between the ages of 30 and 60 (Harcourt 2014). Some studies report a slight female preponderance, and there may be a familial association, with approximately 10% of patients reporting the presence of the disease in a first, second or third degree relative (Requena 2014).

The underlying cause of Ménière's disease is usually not known. Ménière's disease has been associated with an increase in the volume of fluid in the inner ear (endolymphatic hydrops). This may be caused by the abnormal production or resorption of endolymph (Hallpike 1938; Yamakawa 1938). However, it is not clear whether this is the underlying cause of the condition, or merely associated with the disease. Some authors have proposed other underlying causes for Ménière's disease, including viral infections (Gacek 2009), allergic (Banks 2012) or autoimmune disease processes (Greco 2012). A genetic predisposition has also been noted (Chiarella 2015). Occasionally, the symptoms may be secondary to a known cause (such as a head injury or other inner ear disorder) – in these cases it may be referred to as Ménière's syndrome.

Although Ménière's disease is relatively uncommon, it has a profound impact on quality of life. The unpredictable, episodic nature of the condition and severe, disabling attacks of vertigo cause a huge amount of distress. Quality of life (including physical and psychosocial aspects) is significantly reduced for those with Ménière's disease (Söderman 2002). The costs of the condition are also considerable, both in relation to medical interventions (appointments, diagnostic tests and treatments) and loss of productivity or sick days for those affected by the condition (Tyrrell 2016).

### Description of the intervention

A variety of different interventions have been proposed to treat people with Ménière's disease. These include dietary or lifestyle changes, oral treatments, treatments administered by injection into the ear (intratympanic) and surgical treatments. This review focuses on the use of surgical interventions to treat the symptoms of Ménière's disease.

Surgical treatments fall into two main categories ‐ either non‐destructive or destructive. Non‐destructive treatments aim to preserve hearing and sometimes balance function in the ear, and include the following:

- insertion of ventilation tubes;
- endolymphatic sac surgery;
  - endolymphatic sac decompression;
  - insertion of an endolymphatic shunt;
  - blockage or obliteration of the endolymphatic duct;
- semi‐circular canal plugging or obliteration.

Destructive treatments aim to eradicate balance function in the affected ear and may sacrifice remaining hearing, or have other potential serious risks. These include labyrinthectomy or, more selective vestibular nerve section. They are typically used as an intervention of 'last resort' when other interventions have failed to control the symptoms. 

At present, there is no agreement on which is the ideal treatment for people with Ménière's disease – consequently there is no 'gold standard' treatment with which to compare these interventions. 

### How the intervention might work

As the underlying cause of Ménière's disease is poorly understood, so too are the ways in which the interventions may work. 

Some non‐destructive surgical treatments aim to address assumed endolymphatic hydrops, by reducing pressure in the endolymph on the presumption that this will alleviate some of the symptoms of the disease. This is hypothesised to be achieved by creating more space for the endolymph fluid by removing a small amount of bone (decompression), and/or placing a small tube (shunt) to allow the endolymph to drain. A more recent approach has been to occlude/block the endolymphatic duct (Saliba 2015), with a similar aim of reducing endolymph by reducing flow from the endolymphatic sac to the inner ear. 

Some authors have suggested that the middle ear may play a role in Ménière's disease, and consequently have tried inserting ventilation tubes (Tumarkin 1961). However, this has not been widely adopted in the management of Ménière's disease, as the mechanism of action is uncertain. 

Plugging one or more of the semi‐circular canals has also been suggested as a treatment for Ménière's disease (Charpiot 2010). This is on the presumption that stimulation of the semi‐circular canals is responsible for most of the vertigo symptoms during an attack. 

Destructive surgery aims to completely remove unilateral vestibular input. This can be achieved by irreversibly damaging or drilling out the labyrinth (which sacrifices any remaining hearing) or, more selectively, dividing the vestibular nerve. The latter approach aims to preserve the cochlear nerve and hearing, but is associated with greater surgical risk. It is expected that ‐ with no persisting vestibular function ‐ acute vertigo from Ménière's attacks will cease permanently. Whilst patients may suffer a more chronic vertigo/imbalance from reduced unilateral vestibular function, it is usual for this to improve through central compensation. 

### Why it is important to do this review

Balance disorders can be difficult to diagnose and treat. There are few specific diagnostic tests, a variety of related disorders with similar symptoms and a limited number of interventions that are known to be effective. To determine which topics within this area should be addressed with new or updated systematic reviews we conducted a scoping and prioritisation process, involving stakeholders (https://ent.cochrane.org/balance-disorders-ent). Ménière's disease was ranked as one of the highest priority topics during this process (along with vestibular migraine and persistent postural perceptual dizziness). 

Although Ménière's disease is a relatively uncommon condition, the significant impact it has on quality of life demonstrates the clear importance of identifying effective interventions to alleviate the symptoms. There is considerable variation in the management of Ménière's disease on both a national and international scale, with a lack of consensus about appropriate first‐line and subsequent therapies. 

This review is part of a suite of six that consider different interventions for Ménière's disease. Through these reviews, we hope to provide a thorough summary of the efficacy (benefits and harms) of the different treatment options, to support people with Ménière's disease (and healthcare professionals) when making decisions about their care. 

## Objectives

To evaluate the benefits and harms of surgical interventions versus placebo or no treatment in people with Ménière's disease.

## Methods

### Criteria for considering studies for this review

#### Types of studies

We included randomised controlled trials (RCTs) and quasi‐randomised trials (where trials were designed as RCTs, but the sequence generation for allocation of treatment used methods such as alternate allocation, birth dates etc). 

Ménière's disease is known to fluctuate over time, which may mean that cross‐over trials are not an appropriate study design for this condition. However, no cross‐over RCTs or cluster RCTs were identified as relevant for inclusion in this review.

We included studies reported as full‐text, those published as conference abstracts only and unpublished data. 

Ménière's disease is characterised by episodic balance disturbance ‐ the frequency of attacks may change over time (Huppert 2010). For studies to obtain accurate estimates of the effect of different interventions, we consider that follow‐up of participants should be for at least three months ‐ to ensure that participants are likely to have experienced a number of attacks during the follow‐up period. Studies that followed up participants for less than three months were excluded from the review.

#### Types of participants

We included studies that recruited adult participants (aged 18 years or older) with a diagnosis of definite or probable Ménière's disease, according to the agreed criteria of the American Academy Otolaryngology ‐ Head and Neck Surgery (AAO‐HNS), the Japan Society for Equilibrium Research, the European Academy of Otology and Neurotology and the Bárány Society. These criteria are outlined in Appendix 1 and described in Lopez‐Escamez 2015. 

If studies used different criteria to diagnose Ménière's disease, we included them if those criteria were clearly analogous to those described in Lopez‐Escamez 2015. For example, studies that used earlier definitions of Ménière's disease (from the AAO‐HNS guidelines of 1995) were also included.  If there was uncertainty over the criteria used for the study, then we made a decision on whether to include the study. This decision was taken by authors who were masked to other features of the studies (such as study size, other aspects of methodology, results of the study) to avoid the introduction of bias in study selection. If a study was conducted in an ENT department and participants were diagnosed with Ménière's disease then we considered it was likely that other diagnoses had been excluded, and included the study. However, we reflected this uncertainty in diagnosis by considering the study at risk of indirectness when using GRADE to assess the certainty of the evidence (see 'Summary of findings and assessment of certainty of the evidence').  

We anticipated that most studies would include participants with active Ménière's disease. We did not exclude studies if the frequency of attacks at baseline was not reported or was unclear, but we planned to highlight if there were differences between studies that may impact on our ability to pool the data, or affect the applicability of our findings.

We excluded studies where participants had previously undergone destructive/ablative treatment for Ménière's disease in the affected ear (such as vestibular neurectomy, chemical or surgical labyrinthectomy), as we considered that they were unlikely to respond to interventions in the same way as those who had not undergone such treatment.

#### Types of interventions

We included the following interventions:

- Ventilation tubes
- Endolymphatic sac decompression and/or shunt or blockage/obliteration
- Semi‐circular canal plugging/obliteration
- Vestibular nerve section
- Labyrinthectomy

The main comparisons were as follows:

- Ventilation tubes versus placebo/no treatment
- Endolymphatic sac decompression and/or shunt or blockage/obliteration versus placebo/no treatment
- Semi‐circular canal plugging/obliteration versus placebo/no treatment
- Vestibular nerve section versus placebo/no treatment
- Labyrinthectomy versus placebo/no treatment

##### Concurrent treatments

There were no limits on the type of concurrent treatments used, providing these were used equally in each arm of the study. We planned to pool studies that included concurrent treatments with those where participants did not receive concurrent treatment, but this was not necessary. 

#### Types of outcome measures

We assessed outcomes at the following time points: 

- 3 to < 6 months;
- 6 to ≤ 12 months;
- > 12 months.

The exception was for adverse event data, when we used the longest time period of follow‐up. 

We searched the COMET database for existing core outcome sets of relevance to Ménière's disease and vertigo, but were unable to find any published core outcome sets. We therefore conducted a survey of individuals with experience of (or an interest in) balance disorders to help identify the outcomes that should be prioritised. The review author team used the results of this survey to inform the choice of outcome measures in this review. 

We analysed the following outcomes in the review, but did not use them as a basis for including or excluding studies.

##### Primary outcomes

- Improvement in vertigo
  - Measured as a dichotomous outcome (improved/not improved), according to self‐report, or according to a change of a specified score (as described by the study authors) on a vertigo rating scale.
- Change in vertigo
  - Measured as a continuous outcome, to identify the extent of change in vertigo symptoms.
- Serious adverse events
  - Including any event that causes death, is life‐threatening, requires hospitalisation, results in disability or permanent damage, or in congenital abnormality. Measured as the number of participants who experience at least one serious adverse event during the follow‐up period. Where possible, we planned to identify data on the occurrence of the following specified, serious adverse events:
    - cranial neuropathies, including facial paralysis;
    - meningitis.

Vertigo symptoms comprise a variety of different features, including frequency of episodes, duration of episodes and severity/intensity of the episodes. Where possible, we included data for the vertigo outcomes that encompassed all of these three aspects (frequency, duration and severity/intensity of symptoms). However, we anticipated that these data may not be available from all studies. We therefore extracted data on the frequency of vertigo episodes as an alternative measure for these outcomes. 

##### Secondary outcomes

- Disease‐specific health‐related quality of life
  - Measured with the Dizziness Handicap Inventory (DHI, Jacobsen 1990), a validated measurement scale in widespread use. If data from the DHI were unavailable we extracted data from alternative validated measurement scales, according to the order of preference described in the list below (based on the validity of the scales for this outcome):
    - DHI short form (Tesio 1999);
    - DHI screening tool (Jacobsen 1998);
    - Vertigo Handicap Questionnaire (Yardley 1992);
    - Meniere's Disease Patient Oriented Symptoms Inventory (MD POSI, Murphy 1999);
    - University of California Los Angeles Dizziness Questionnaire (UCLADQ, Honrubia 1996);
    - AAO‐HNS Functional Level Scale (FLS, AAO‐HNS 1995).
- Hearing
  - Measured with pure tone audiometry and reported as the change in pure tone average (PTA), or (alternatively) by patient report, if data from PTA were not available.
- Tinnitus
  - Measured using any validated, patient‐reported questionnaire relating to the impact of tinnitus, for example the Tinnitus Handicap Inventory (THI, Newman 1996) or the Tinnitus Functional Index (TFI, Meikle 2012). 
- Other adverse effects
  - Measured as the number of participants who experienced at least one episode of the specified adverse effects during the follow‐up period. Including the following specified adverse effects:
    - cerebrospinal fluid (CSF) leak;
    - otitis media;
    - total hearing loss (this is relevant for non‐destructive interventions only, as destructive interventions will always cause total hearing loss in the affected ear).

### Search methods for identification of studies

The Cochrane Ear, Nose and Throat Disorders Group (CENTDG) Information Specialist conducted systematic searches for randomised controlled trials and controlled clinical trials in October 2021 and September 2022. There were no language, publication year or publication status restrictions. The date of the latest search was 14 September 2022.

#### Electronic searches

The Information Specialist searched:

- the Cochrane ENT Trials Register (search via the Cochrane Register of Studies to 14 September 2022);
- the Cochrane Central Register of Controlled Trials (CENTRAL) (search via the Cochrane Register of Studies to 14 September 2022);
- Ovid MEDLINE(R) Epub Ahead of Print, In‐Process & Other Non‐Indexed Citations, Ovid MEDLINE(R) Daily and Ovid MEDLINE(R) (1946 to 14 September 2022);
- Ovid Embase (1974 to 14 September 2022);
- Web of Knowledge, Web of Science (1945 to 14 September 2022);
- ClinicalTrials.gov, www.clinicaltrials.gov (to 14 September 2022);
- World Health Organization (WHO) International Clinical Trials Registry Platform (ICTRP), https://trialsearch.who.int/ (to 14 September 2022).

The Information Specialist modelled subject strategies for databases on the search strategy designed for CENTRAL. The strategy has been designed to identify all relevant studies for a suite of reviews on various interventions for Ménière's disease. Where appropriate, they were combined with subject strategy adaptations of the highly sensitive search strategy designed by Cochrane for identifying randomised controlled trials and controlled clinical trials (as described in the *Cochrane Handbook for Systematic Reviews of Interventions* Version 5.1.0, Box 6.4.b, Handbook 2011). Search strategies for major databases including CENTRAL are provided in Appendix 2.

#### Searching other resources

We scanned the reference lists of identified publications for additional trials and contacted trial authors where necessary. In addition, the Information Specialist searched Ovid MEDLINE to retrieve existing systematic reviews relevant to this systematic review, so that we could scan their reference lists for additional trials. In addition, the Information Specialist ran a non‐systematic search of Google Scholar to identify trials not published in mainstream journals. 

We did not perform a separate search for adverse effects. We considered adverse effects described in included studies only.

### Data collection and analysis

#### Selection of studies

The Cochrane ENT Information Specialist used the first two components of Cochrane's Screen4Me workflow to help assess the search results: 

1. Known assessments – a service that matches records in the search results to records that have already been screened in Cochrane Crowd and been labelled as 'a RCT' or as 'not a RCT'. 
2. The machine learning classifier (RCT model) (Wallace 2017), available in the Cochrane Register of Studies (CRS‐Web), which assigns a probability of being a true RCT (from 0 to 100) to each citation. Citations that were assigned a probability score below the cut‐point at a recall of 99% were assumed to be non‐RCTs. We manually dual screened the results for those that scored on or above the cut‐point. 

At least two review authors (of BG, AL, KW) or co‐workers (KG and SC, listed in Acknowledgements) independently screened the remaining titles and abstracts using Covidence, to identify studies that may be relevant for the review. Any discrepancies were resolved by consensus, or by retrieving the full text of the study for further assessment. 

We obtained the full text for any study that was considered possibly relevant and two authors (of BG, AL, KW) or a co‐worker (KG) again independently checked this to determine whether it met the inclusion criteria for the review. Any differences were resolved by discussion and consensus, or through recourse to a third author if necessary. 

We excluded any studies that were retrieved in full text but subsequently deemed to be inappropriate for the review (according to the inclusion/exclusion criteria), according to the main reason for exclusion. 

The unit of interest for the review is the study, therefore multiple papers or reports of a single study are grouped together under a single reference identification. The process for study selection is recorded in Figure. 

**1:**
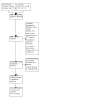
Flow chart of study retrieval and selection.

##### Screening eligible studies for trustworthiness

We assessed studies meeting our inclusion criteria for trustworthiness using a screening tool developed by Cochrane Pregnancy and Childbirth. This tool includes specified criteria to identify studies that are considered sufficiently trustworthy to be included in the review (see Appendix 3 and Figure). If studies were assessed as being potentially 'high‐risk', we attempted to contact the study authors to obtain further information or address any concerns. We planned to exclude studies from the main analyses of the review if there were persisting concerns over trustworthiness, or we were unable to contact the authors. 

**2:**
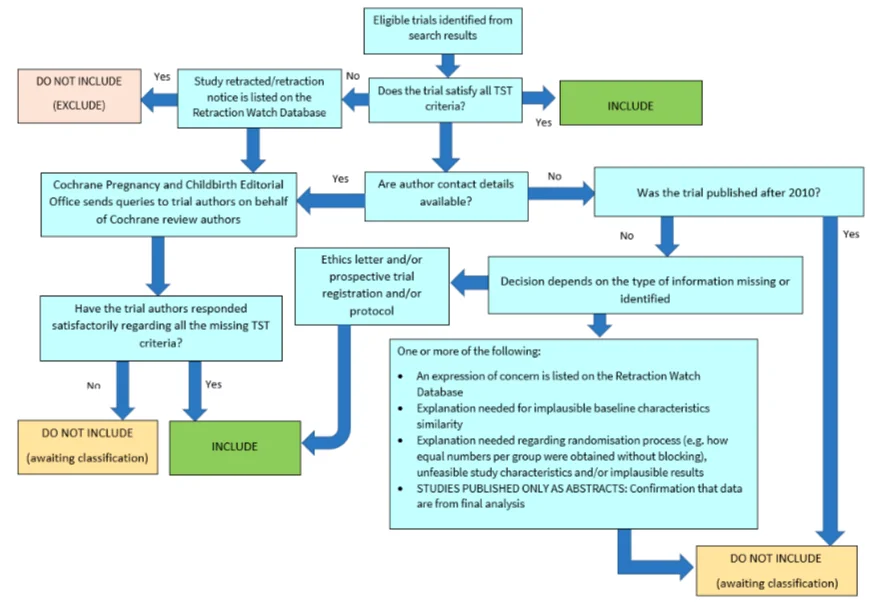
The Cochrane Pregnancy and Childbirth Trustworthiness Screening Tool

However, both of the included studies had some concerns when using the tool. For Kitahara 2016, this was due to the large effect size seen for the primary outcome measures, despite the relatively small size of the trial, and also because only limited baseline information was reported (preventing full assessment of the randomisation process). For Thomsen 1981 we had some concerns over the randomisation process, due to the equal numbers of participants (and gender balance) in the groups, despite no report of stratified randomisation. 

We had not anticipated this issue when drafting the protocol for our review (Webster 2021b), but it is likely to be a widespread issue for reviews that incorporate older studies, and has been a persistent problem through this suite of reviews on Ménière's disease.

There are several possible explanations for the large number of studies across the suite that had concerns when using the tool. One is that there are issues with the trustworthiness of the studies identified in this review, and the data included may not give reliable estimates of the true effect. Alternatively, the trustworthiness screening tool may be excessively sensitive, and flag studies that are trustworthy, but where information has not been fully reported. We note that this tool (and others used for the same purpose) has not yet been validated for use. 

We therefore took the decision to include the studies in the review, despite the potential concerns over trustworthiness. The uncertainty in the results is captured as part of our GRADE rating in the certainty of the evidence, using the domain 'study limitations'. 

#### Data extraction and management

Two review authors (of BG, AL, KW) independently extracted outcome data from each study using a standardised data collection form. Where a study had more than one publication, we retrieved all publications to ensure that we had a complete data set. We checked any discrepancies in the data extracted by the two authors against the original reports, and resolved differences through discussion and consensus. If required, we contacted the study authors for clarification.

We extracted data on the key characteristics of the studies, including the following information:

- study design, duration of the study, number of study centres and location, study setting and dates of the study;
- information on the participants, including the number randomised, those lost to follow‐up or withdrawn, the number analysed, the age of participants, gender, severity of the condition, diagnostic criteria used, inclusion and exclusion criteria for the individual studies;
- details of the intervention, comparator, and concomitant treatments or excluded medications;
- the outcomes specified and reported by the study authors, including the time points;
- funding for the study and any conflicts of interest for the study authors;
- information required to assess the risk of bias in the study, and to enable GRADE assessment of the evidence.

Once the extracted data were checked and any discrepancies resolved, a single author (KW) transferred the information to Review Manager 5 (RevMan 2020).

The primary effect of interest for this review is the effect of treatment assignment (which reflects the outcomes of treatment for people who were assigned to the intervention) rather than a per protocol analysis (the outcomes of treatment only for those who completed the full course of treatment as planned). For the outcomes of interest in this review, we extracted the findings from the studies on an available case basis, i.e. all available data from all participants at each time point, based on the treatment to which they were randomised. This was irrespective of compliance, or whether participants had received the intervention as planned.

In addition to extracting pre‐specified information about study characteristics and aspects of methodology relevant to risk of bias, we extracted the following summary statistics for each study and outcome:

- For continuous data: the mean values, standard deviation and number of patients for each treatment group at the different time points for outcome measurement. Where change‐from‐baseline data were not available, we extracted the values for endpoint data instead. If values for the individual treatment groups were not reported, where possible we extracted summary statistics (e.g. mean difference) from the studies.
- For binary data: we extracted information on the number of participants experiencing an event, and the number of participants assessed at that time point. If values for the individual treatment groups were not reported, where possible we extracted summary statistics (e.g. risk ratio) from the studies.
- For ordinal scale data: if the data appeared to be normally distributed, or if the analysis performed by the investigators indicated that parametric tests are appropriate, then we treated the outcome measure as continuous data. 
- For time‐to‐event data: we did not identify any time‐to‐event data for the outcomes specified in the review. 

If necessary, we converted data found in the studies to a format appropriate for meta‐analysis, according to the methods described in the *Cochrane Handbook for Systematic Reviews of Interventions* (Handbook 2011). 

We pre‐specified time points of interest for the outcomes in this review. Where studies reported data at multiple time points, we planned to take the longest available follow‐up point within each of the specific time frames. The study Thomsen 1981 reported at multiple time points over a nine‐year period. Due to high attrition over the course of the study, we have included the results at these different times where possible. 

#### Assessment of risk of bias in included studies

Two authors (KW and AL) undertook assessment of the risk of bias of the included studies independently, with the following taken into consideration, as guided by the *Cochrane Handbook for Systematic Reviews of Interventions* (Handbook 2011):

- sequence generation;
- allocation concealment;
- blinding;
- incomplete outcome data;
- selective outcome reporting; and
- other sources of bias.

We used the Cochrane risk of bias tool (Handbook 2011), which involves describing each of these domains as reported in the study and then assigning a judgement about the adequacy of each entry: 'low', 'high' or 'unclear' risk of bias.

#### Measures of treatment effect

We summarised the effects of the majority of dichotomous outcomes (e.g. serious adverse effects) as risk ratios (RR) with 95% confidence intervals (CIs). We have also expressed the results as absolute numbers based on the pooled results and compared to the assumed risk in the summary of findings tables (Table; Table) and full GRADE profiles (Table; Table). 

**1:** GRADE profile: Ventilation tubes compared to no treatment for Ménière's disease

**Certainty assessment**							**№ of participants**		**Effect**		**Certainty**	**Comment**
**№ of studies**	**Study design**	**Risk of bias**	**Inconsistency**	**Indirectness**	**Imprecision**	**Other considerations**	**Ventilation tubes**	**No treatment**	**Relative** **(95% CI)**	**Absolute** **(95% CI)**		
**Improvement in frequency of vertigo (complete resolution) at > 12 months**												
1	Randomised trials	Serious^a,b^	Not serious	Serious^c^	Serious^d^	None	53/63 (84.1%)	38/70 (54.3%)	**RR 1.55** (1.22 to 1.97)	**299 more per 1000** (from 119 more to 527 more)	⨁◯◯◯ Very low	The evidence is very uncertain about the effect of ventilation tubes on improvement in vertigo frequency at > 12 months.
**Change in hearing: improvement in hearing at > 12 months**												
1	Randomised trials	Serious^a^	Not serious	Not serious	Serious^d^	None	22/63 (34.9%)	5/70 (7.1%)	**RR 4.89** (1.97 to 12.14)	**278 more per 1000** (from 69 more to 796 more)	⨁⨁◯◯ Low	Ventilation tubes may increase the proportion of people who experience improvement in hearing at > 12 months.

**CI:** confidence interval; **RR:** risk ratio ^a^Unblinded study. ^b^Risk of selective reporting for this outcome. ^c^Outcome considers 'complete resolution' of vertigo only, not 'any improvement'. ^d^Optimal information size was not reached, taken as > 300 events for dichotomous outcomes, as a rule of thumb.

**2:** GRADE profile: Endolymphatic sac decompression compared to sham surgery for Ménière’s disease

**Certainty assessment**							**№ of participants**		**Effect**		**Certainty**	**Comment**
**№ of studies**	**Study design**	**Risk of bias**	**Inconsistency**	**Indirectness**	**Imprecision**	**Other considerations**	**Endolymphatic sac decompression**	**Sham surgery**	**Relative** **(95% CI)**	**Absolute** **(95% CI)**		
**Improvement in vertigo (complete resolution) at 6 to ≤ 12 months (12 months)**												
1	Randomised trials	Serious^a^	Not serious	Serious^b,c ^	Very serious^d^	None	13/15 (86.7%)	10/15 (66.7%)	**RR 1.30** (0.86 to 1.96)	**200 more per 1000** (from 93 fewer to 640 more)	⨁◯◯◯ Very low	The evidence is very uncertain about the effect of endolymphatic sac surgery on improvement in vertigo at 6 to ≤ 12 months.
**Improvement in vertigo (complete resolution) at > 12 months (9 years)**												
1	Randomised trials	Serious^a^	Not serious	serious^b,c^	Very serious^d^	None	10/11 (90.9%)	12/12 (100.0%)	**RR 0.91** (0.72 to 1.16)	**90 fewer per 1000** (from 280 fewer to 160 more)	⨁◯◯◯ Very low	The evidence is very uncertain about the effect of endolymphatic sac surgery on improvement in vertigo at 9 years.
**Change in vertigo (assessed with: daily rating scale; scale from: 0 to 93, higher scores = worse symptoms) at 6 to ≤ 12 months (12 months)**												
1	Randomised trials	Serious^a^	Not serious	Serious^e^	Very serious^d^	None	14	15	—	MD **12.39 points lower** (22.81 lower to 1.97 lower)	⨁◯◯◯ Very low	The evidence is very uncertain about the effect of endolymphatic sac surgery on vertigo frequency and severity at 6 to ≤ 12 months.
**Change in hearing (assessed with PTA) at 6 to ≤ 12 months (12 months)**												
1	Randomised trials	Serious^a^	Not serious	Serious^c^	Very serious^d^	None	15	15	—	MD **8.41 dB HL lower** (17 lower to 0.18 higher)	⨁◯◯◯ Very low	The evidence is very uncertain about the effect of endolymphatic sac surgery on hearing at 6 to ≤ 12 months.
**Change in hearing (assessed with PTA) at > 12 months (9 years)**												
1	Randomised trials	Serious^a^	Not serious	Serious^c^	Very serious^d^	None	15	15	—	MD **2.33 dB HL higher** (16.54 lower to 21.20 higher)	⨁◯◯◯ Very low	The evidence is very uncertain about the effect of endolymphatic sac surgery on hearing at > 12 months.

**CI:** confidence interval; **dB HL:** decibels hearing level; **MD:** mean difference; **PTA:** pure tone audiometry; **RR:** risk ratio ^a^Multiple domains were rated at unclear risk of bias. Re‐analysis of the original trial data by Welling 2000 indicates that the analysis used may have been inappropriate. Later time points (after 12 months) are also affected by attrition bias. ^b^Outcome assessed is 'complete resolution' of vertigo, not any improvement. ^c^The criteria used for the diagnosis of Ménière's disease were poorly defined, therefore the population may not be appropriate. ^d^Sample size is extremely small. Fails to meet optimal information size and confidence intervals are very wide. ^e^An unvalidated scale was used to assess vertigo.

For continuous outcomes, we expressed treatment effects as a mean difference (MD) with standard deviation (SD). We did not need to use the standardised mean difference to pool any data. 

#### Unit of analysis issues

Ménière's disease is unlikely to be a stable condition, and interventions may not have a temporary effect. If cross‐over trials are identified then we planned to use the data from the first phase of the study only. If cluster‐randomised trials were identified then we would have ensured that analysis methods were used to account for clustering in the data (Handbook 2021). However, we did not identify any cross‐over studies, or cluster‐randomised trials.

We did identify one study with four arms (Kitahara 2016). One intervention arm was relevant for this review (ventilation tubes), and is compared to the arm that received no treatment. The remaining arms comprised two active interventions that were not relevant for this review and are included in a separate review of lifestyle and dietary interventions for Ménière's disease (Webster 2022).

#### Dealing with missing data

We planned to contact study authors via email whenever the outcome of interest was not reported, if the methods of the study suggest that the outcome had been measured. We did the same if not all data required for meta‐analysis were reported (for example, standard deviations), unless we were able to calculate them from other data reported by the study authors. 

#### Assessment of heterogeneity

We planned to assess clinical heterogeneity by examining the included studies for potential differences between studies in the types of participants recruited, interventions or controls used and the outcomes measured. However, no meta‐analysis was conducted in the course of this review.

#### Assessment of reporting biases

We assessed reporting bias as within‐study outcome reporting bias and between‐study publication bias.

##### Outcome reporting bias (within‐study reporting bias)

We assessed within‐study reporting bias by comparing the outcomes reported in the published report against the study protocol or trial registry, whenever this could be obtained. If the protocol or trial registry entry was not available, we compared the outcomes reported to those listed in the methods section. If results are mentioned but not reported adequately in a way that allows analysis (e.g. the report only mentions whether the results were statistically significant or not), bias in a meta‐analysis is likely to occur. We then sought further information from the study authors. If no further information was found, we noted this as being a 'high' risk of bias with the risk of bias tool. If there was insufficient information to judge the risk of bias we noted this as an 'unclear' risk of bias (Handbook 2011). 

##### Publication bias (between‐study reporting bias)

We did not have sufficient studies to create funnel plots for any analysis. Any studies identified through trial registries and other sources (Searching other resources) that remain unpublished are noted in the Ongoing studies section. 

#### Data synthesis

##### Meta‐analysis of numerical data

We planned to conduct a meta‐analysis of numerical data where possible and appropriate (if participants, interventions, comparisons and outcomes are sufficiently similar in the trials identified). However, we only identified a single study for each comparison in this review, therefore no meta‐analysis was possible. 

Improvement in vertigo symptoms may be assessed using a variety of methods, which consider different aspects of vertigo. These include:

- frequency of vertigo episodes;
- duration of vertigo episodes;
- severity/intensity of vertigo episodes;
- a composite measure of all of these aspects:
  - for example, assessed with a global score ‐ such as "how troublesome are your vertigo symptoms?", rated on an ordinal scale.

For the outcomes "improvement in vertigo" and "change in vertigo", we prioritised outcome measures that used a composite score ‐ encompassing aspects of vertigo frequency, duration and severity/intensity. Examples of this would include a global rating scale of vertigo impact (rated from 0 to 10, where 0 is defined as no symptoms, and 10 is defined as the most troublesome symptoms) or the vertigo/balance subscale of the Vertigo Symptom Scale (Yardley 1992), or Vertigo Symptom Scale Short Form (Yardley 1998). As data from composite scores were not available from the majority of studies, we also included data on the frequency of vertigo episodes as an alternative measure.

##### Synthesis using other methods

If we were unable to pool numerical data in a meta‐analysis for one or more outcomes we planned to provide a synthesis of the results using alternative methods, following the guidance in Chapter 12 of the Handbook 2021). However, this was not necessary, as results were provided by a single study. 

#### Subgroup analysis and investigation of heterogeneity

If statistical heterogeneity was identified for any comparisons, we planned to assess this considering the following subgroups:

- Different surgical techniques
- Use of concomitant treatment.
- Diagnosis of Ménière's disease

However, due to the paucity of data available, we did not carry out any subgroup analysis. 

#### Sensitivity analysis

We planned to carry out a number of sensitivity analyses for the primary outcomes in this review. However, the paucity of data and the lack of meta‐analyses has meant that this was not possible. 

We intended to carry out sensitivity analyses for the primary outcomes only, considering:

- the use of a fixed‐effect model instead of a random‐effects model;
- the diagnostic criteria used for Ménière's disease;
- the inclusion/exclusion of studies with identified concerns using the Cochrane Pregnancy and Childbirth Group Screening Tool.

#### Summary of findings and assessment of the certainty of the evidence

Two independent authors (AL, KW) used the GRADE approach to rate the overall certainty of evidence using GRADEpro GDT (https://gradepro.org/) and the guidance in chapter 14 of the *Cochrane Handbook for Systematic Reviews of Interventions* (Handbook 2021). Disagreements were resolved through discussion and consensus. The certainty of evidence reflects the extent to which we are confident that an estimate of effect is correct, and we have applied this in the interpretation of results. There are four possible ratings: high, moderate, low and very low. A rating of high certainty of evidence implies that we are confident in our estimate of effect and that further research is very unlikely to change our confidence in the estimate of effect. A rating of very low certainty implies that any estimate of effect obtained is very uncertain.

The GRADE approach rates evidence from RCTs that do not have serious limitations as high certainty. However, several factors can lead to the downgrading of the evidence to moderate, low or very low. The degree of downgrading is determined by the seriousness of these factors:

- Study limitations (risk of bias):
  - This was assessed using the rating from the Cochrane risk of bias tool for the study or studies included in the analysis. We rated down either one or two levels, depending on the number of domains that had been rated at high or unclear risk of bias. 
- Inconsistency:
  - This was assessed using the I^2^ statistic and the P value for heterogeneity for all meta‐analyses, as well as by visual inspection of the forest plot. For results based on a single study we rated this domain as no serious inconsistency.
- Indirectness of evidence:
  - We took into account whether there were concerns over the population included in the study or studies for each outcome, as well as whether additional treatments were offered that may impact on the efficacy of the intervention under consideration. 
- Imprecision:
  - We took into account the sample size and the width of the confidence interval for each outcome. If the sample size did not meet the optimal information size (i.e. < 400 people for continuous outcomes or < 300 events for dichotomous outcomes), or the confidence interval crossed the small effect threshold we rated down one level. If the sample size did not meet the optimal information size and the confidence interval includes both potential harm and potential benefit we rated down twice. We also rated down twice for very tiny studies (e.g. 10 to 15 participants in each arm), regardless of the estimated confidence interval.
- Publication bias:
  - We considered whether there were likely to be unpublished studies that may impact on our confidence in the results obtained. 

We used a minimally contextualised approach, and rated the certainty in the interventions having an important effect (Zeng 2021). Where possible, we used agreed minimally important differences (MIDs) for continuous outcomes as the threshold for an important difference. Where no MID was identified, we provide an assumed MID based on agreement between the authors. For dichotomous outcomes, we looked at the absolute effects when rating imprecision, but also took into consideration the GRADE default approach (rating down when a RR crosses 1.25 or 0.80). We have justified all decisions to downgrade the certainty of the evidence using footnotes, and added comments to aid the interpretation of the findings, where necessary. 

We planned to provide a summary of findings tables for the following comparisons:

- ventilation tubes versus placebo/no treatment;
- endolymphatic sac decompression and/or shunt or blockage/obliteration versus placebo/no treatment;
- semi‐circular canal plugging versus placebo/no treatment.

We have included all primary outcomes in the summary of findings tables. We planned to prioritise outcomes at the time point three to six months for presentation. However, as no data were obtained for this time period we have included data from later time points in the tables. 

## Results

### Description of studies

#### Results of the search

The searches in September 2022 retrieved a total of 4434 records. This reduced to 3408 after the removal of duplicates. The Cochrane ENT Information Specialist sent all 3408 records to the Screen4Me workflow. The Screen4Me workflow identified 122 records as having previously been assessed: 83 had been rejected as not RCTs and 39 had been assessed as possible RCTs. The RCT classifier rejected an additional 1427 records as not RCTs (with 99% sensitivity). We did not send any records to the Cochrane Crowd for assessment. Following this process, the Screen4Me workflow had rejected 1510  records and identified 1898 possible RCTs for title and abstract screening. 

**Table:** 

** **	**Possible RCTs**	**Rejected**
Known assessments	39	83
RCT classifier	1859	1427
Total	1898	1510

We identified 89 additional duplicates. We screened the titles and abstracts of the remaining 1809 records. We discarded 1775 records and assessed 34 full‐text records. 

We excluded 23 records (linked to 19 studies) with reasons recorded in the review (see Excluded studies). We included two completed studies (10 records) where results were available. One record is listed as an ongoing study (see Characteristics of ongoing studies for details).

A flow chart of study retrieval and selection is provided in Figure.

#### Included studies

We included two RCTs (Kitahara 2008; Thomsen 1981). Details of individual studies can be found in the Characteristics of included studies.

##### Study design

Both studies were described as randomised controlled trials. The duration of initial follow‐up for Thomsen 1981 was one year; however, the authors subsequently published a series of articles with extended follow‐up for up to nine years for some participants. Kitahara 2016 followed up participants for 24 months. 

##### Participants

All the included studies recruited adult participants with a diagnosis of Ménière's disease.

###### Diagnosis of Ménière's disease

- Thomsen 1981 reported that participants had typical attacks of fluctuating hearing loss, tinnitus and vertigo often accompanied by nausea, vomiting and pressure in the ear with at least one attack every two weeks. However, there was no mention of the use of specific criteria to diagnose Ménière's disease.
- Kitahara 2016 reported that participants fulfilled the AAO‐HNS 1995 criteria for definite Ménière's disease. 

###### Features of Ménière's disease

Thomsen 1981 reported that five out of 30 patients had bilateral disease. Participants had symptoms for at least six months, but no longer than five years.

Kitahara 2016 did not state whether participants had unilateral or bilateral disease. No information was provided regarding the duration of Ménière's symptoms. Participants had an average of 1.7 vertigo attacks per month at baseline. 

Both studies indicated that participants had some form of medical treatment before entering the trial. Thomsen 1981 recruited those who underwent unsuccessful medical treatment. However, this study did not provide information as to which treatments they received nor information regarding adjuvant therapy during the trial. Participants in Kitahara 2016 had three to six months of "fixed forms of medical treatment", but did not have sufficient benefit from this. They included diuretics, betahistine, diphenidol, dimenhydrinate and diazepam.

##### Interventions and comparisons

The studies considered two of our proposed comparison pairs. No studies were identified that considered the use of semi‐circular canal plugging/obliteration, vestibular nerve section or labyrinthectomy.

###### Comparison 1: Ventilation tubes versus no intervention (plus medical therapy for both groups)

Kitahara 2016 compared the insertion of ventilation tubes under local anaesthesia (alongside standard medical therapy) to standard medical therapy alone for Ménière's disease. The type of ventilation tube used was not reported. All participants in the trial received "standard medical therapy". The authors provided this statement: "Medical treatments were fixed basically including diuretics, betahistine, diphenidol, dimenhydrinate, and diazepam, all considered effective for persistent symptoms of Meniere’s disease". We presume that a selection from these medications was tailored to each individual in the trial, but this is not clearly reported. 

###### Comparison 2: Endolymphatic sac surgery versus placebo (sham surgery)

This comparison was assessed in Thomsen 1981. Participants underwent a standard endolymphatic sac shunt operation with insertion of Silastic into the sac, which drained out into the mastoid cavity. Those in the control group underwent sham surgery, and received a standard mastoidectomy operation "at which much care was taken not to remove the bone over the endolymphatic sac, in order to avoid a decompression". Although the control group received an intervention, it was agreed that this was unlikely to have any impact on symptoms of Ménière's disease, therefore was an appropriate "placebo" control for this comparison. 

##### Outcomes

###### 1. Improvement in vertigo

For this outcome we included dichotomous data ‐ assessed as the proportion of participants whose vertigo had 'improved' or 'not improved'. 

####### 1.1. Global score

Neither study reported the improvement of vertigo using a global score that considered the frequency, duration and intensity of vertigo attacks. 

####### 1.2. Frequency

Kitahara 2016 used the AAO‐HNS 1995 criteria to define a vertigo attack. They then devised a ratio of pre‐post treatment to determine the rate of vertigo control. The pre‐treatment frequency of vertigo was defined as the number of attacks during the six months before admission to their study, and post‐treatment frequency of vertigo as the number of attacks between 18 and 24 months afterwards. A score of < 0.8 was regarded as improvement. However, the article only reports the proportion of participants who had *complete resolution* of vertigo, not the proportion who experienced any improvement.

Thomsen 1981 reported their results according to the AAOO 1972 criteria for improvement. This considers vertigo and hearing, but classifies improvement in vertigo as either complete resolution or continued vertigo.

###### 2. Change in vertigo

This outcome included data on the change in vertigo using a continuous numerical scale. 

####### 2.1. Global score

Kitahara 2016 did not consider the change in vertigo using a global score.

Thomsen 1981 used a three‐point scale to classify patients' dizziness and vertigo symptoms. The scoring was as follows: "0 = no dizziness/vertigo; 1 = weak‐rare, noted occasionally; 2 = strong‐significant, almost all of the time but tolerable; 3 = severe, interferes with daily activities, patient stays in bed". The median monthly score over the follow‐up time was then reported, giving a range of scores from 0 to 93, with higher scores representing worse symptoms. This scoring system would presumably encompass the frequency, duration and severity of vertigo episodes. It is not known if these scales were previously validated.

###### 3. Serious adverse events

These were not reported by either of the included studies. 

###### 4. Disease‐specific health‐related quality of life

Neither study considered disease‐specific health‐related quality of life using a scale that specifically assessed the impact of dizziness or vertigo. 

###### 5. Hearing

Kitahara 2016 used the AAO‐HNS 1995 criteria to evaluate by measuring pure tone audiometry based on a four tone average using 0.5 kHz, 1 kHz, 2 kHz and 4 kHz. The worst hearing level recorded during the six months before admission to their study was used as the pre‐treatment level and the worst hearing level between 18 and 24 months as the post‐treatment hearing level.

Thomsen 1981 assessed hearing using pure tone audiometry at 0.25 kHz, 0.5 kHz and 1 kHz.

###### 6. Tinnitus

Neither study assess tinnitus using a validated scale that considered the impact of tinnitus on quality of life. Kitahara 2016 did not have tinnitus as a outcome. Thomsen 1981 only assessed tinnitus using a composite scale (combined with hearing loss, dizziness, nausea/vomiting and aural pressure). 

###### 7. Other adverse effects

Neither study reported on the specified adverse events of interest in this review (CSF leak, otitis media or total hearing loss).

#### Excluded studies

After assessing the full text, we excluded 19 studies (linked to 23 records) from this review. The main reason for the exclusion of each study is listed below.

Four studies were not randomised controlled trials (Arslan 1971; Heatley 1991; Sakagami 2015; UMIN000005544).

Three studies were narrative review articles on Ménière's disease and did not report any primary data (Beck 1985; Conde Jahn 1965; Richards 1971).

Ten studies were RCTs but did not compare an active intervention to no treatment or placebo (Bojrab 2018; Kitahara 2008; Lu 2002; NCT01574313; NCT04847700; Saliba 2015; Schenck 2021; Thomsen 1998; Welling 1996; Xu 2022). Most of these compared two different types of surgical technique. The study Thomsen 1998 included two of our interventions of interest (endolymphatic sac surgery and ventilation tubes) but compared them to each other ‐ no control arm was included. Therefore this study was not eligible for inclusion in the review. 

Two studies were critiques or re‐analysis of the study Thomsen 1981 but did not report any primary data (Arenberg 1981; Welling 2000).

### Risk of bias in included studies

See Figure for the risk of bias summary (our judgements about each risk of bias item for each included study) and Figure for the risk of bias graph (our judgements about each risk of bias item presented as percentages across all included studies). Both studies had some concerns regarding the risk of bias, with at least two domains being rated at high risk of bias.

**3:**
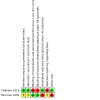
Risk of bias summary (our judgements about each risk of bias item for each included study).

**4:**
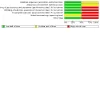
Risk of bias graph (our judgements about each risk of bias item presented as percentages across all included studies).

#### Allocation

Kitahara 2016 provided sufficient details on the methods used for randomisation as well as allocation concealment. Therefore we rated it at low risk. Thomsen 1981 did not describe the methods used for randomisation and allocation concealment. We therefore rated it as unclear risk.

#### Blinding

Kitahara 2016 was an open‐labelled study. Both participants and study personnel were aware of the group allocation. We rated it as high risk. Thomsen 1981 provided details on how the investigators were deprived of any information about the operative procedure employed. In subsequent iterations of this study the participants were still unaware of which kind of surgery was carried out. We rated it as low risk.

#### Incomplete outcome data

We rated Kitahara 2016 as low risk for this domain as the number of dropouts was low (< 10%) and balanced across the groups. Thomsen 1981 maintained full follow‐up until 12 months. Thereafter there was considerable loss to follow‐up by nine years. We therefore rated it as high risk overall for this domain. However, we acknowledge that the risk of bias at earlier time points (up to 12 months follow‐up) is low.

#### Selective reporting

Kitahara 2016 used a ratio of pre/post‐treatment values to define vertigo control as "better", "worse" or "no change". However, the authors only report the number of participants in whom vertigo resolved completely over the course of the study. Although the study was prospectively registered with ClinicalTrials.gov, the only outcome listed here is "all cause mortality". This seems a surprising choice of primary outcome measure (and is also not reported in the publication). There was no report of any adverse events, including relatively common complications of ventilation tube placement, such as otorrhoea or tympanic membrane perforation. We rated it as high risk.

Thomsen 1981 did not publish a protocol. It was unclear if all outcomes are fully reported. Also, there were no adverse events published at the 12‐month follow‐up. We rated it as unclear risk.

#### Other potential sources of bias

No additional sources of bias were identified in Kitahara 2016.

We are aware that the data presented in Thomsen 1981 were subsequently re‐analysed leading to different conclusions (Welling 2000). Also, there was no control around how patients were treated medically during the nine‐year follow‐up study, which could have a significant impact on the results. We rated it as high risk.

### Effects of interventions

See: **Summary of findings 1** Ventilation tubes compared to no treatment for Ménière’s disease; **Summary of findings 2** Endolymphatic sac decompression compared to placebo (sham surgery) for Ménière’s disease

#### 1. Ventilation tubes compared to no intervention (plus medical therapy for both groups)

This comparison was addressed by Kitahara 2016. All follow‐up was conducted at 24 months.

##### 1.1. Improvement in vertigo

###### 1.1.1. Global score

This study did not consider improvement in vertigo using a global score.

###### 1.1.2. Vertigo frequency

####### 1.1.2.1. 3 to < 6 months

The study did not report at this time point. 

####### 1.1.2.2. 6 to ≤ 12 months

The study did not report at this time point. 

####### 1.1.2.3. > 12 months

The risk ratio (RR) for improvement in the frequency of vertigo (complete resolution) for those receiving ventilation tubes was 1.55 (95% confidence interval (CI) 1.22 to 1.97; 1 study; 133 participants; Analysis 1.1; very low‐certainty evidence).

##### 1.2. Change in vertigo

This outcome was not reported. 

##### 1.3. Serious adverse events

No serious adverse events were reported. It is unclear whether this is because they did not occur, or because the authors did not monitor and report adverse events. 

##### 1.4. Disease‐specific health‐related quality of life

This outcome was not reported. 

##### 1.5. Change in hearing

Kitahara 2016 measured the hearing results using the AAO‐HNS 1995 criteria based on a four‐tone average at 0.5 kHz, 1 kHz, 2 kHz and 4 kHz. The worst hearing level during the six months before admission to this study was considered the pre‐treatment hearing level, and the worst hearing level between 18 and 24 months the post‐treatment hearing level. More than 10 dB differences in hearing levels before and after treatment were regarded as "better", less than 10 dB differences as "worse" and the remainder as "no change".

###### 1.5.1. 3 to < 6 months

The study did not report at this time point. 

###### 1.5.2. 6 to ≤ 12 months

The study did not report at this time point. 

###### 1.5.3. > 12 months

The RR for improvement in hearing at 24 months was 4.89 (95% CI 1.97 to 12.14; 1 study; 133 participants; Analysis 1.2; low‐certainty evidence).

##### 1.6. Change in tinnitus

This outcome was not reported. 

##### 1.7. Other adverse effects

No adverse effects were reported. As above, it is unclear whether this is because they did not occur, or because the authors did not monitor and report adverse effects. 

#### 2. Endolymphatic sac decompression compared to placebo (sham surgery) for Ménière’s disease

One study addressed this comparison (Thomsen 1981). 

##### 2.1. Improvement in vertigo

For this outcome we included any data that were reported as a dichotomous (binary) outcome ‐ i.e. classifying participants as having improved or not improved. 

###### 2.1.1. Global score

Thomsen 1981 did not consider improvement in vertigo using a global score, which included frequency, duration and severity of vertigo. 

###### 2.1.2. Vertigo frequency

Thomsen 1981 used the AAOO 1972 criteria for vertigo control, which classify individuals as having complete resolution of vertigo, or ongoing symptoms.

####### 2.1.2.1. At 3 to < 6 months

No data were reported at this time point.

####### 2.1.2.2. At 6 to ≤ 12 months

The risk ratio for improvement (complete resolution of vertigo) was 1.30 in those receiving endolymphatic sac decompression (95% CI 0.86 to 1.96; 1 study; 30 participants; Analysis 2.1; very low‐certainty evidence).

####### 2.1.2.3. At > 12 months

The risk ratio for improvement (complete resolution) at nine years was 0.91 (95% CI 0.72 to 1.16; 1 study; 23 participants; Analysis 2.1; very low‐certainty evidence).

##### 2.2. Change in vertigo

For this outcome we included any continuous data ‐ where the change in vertigo was measured on a continuous scale (such as with a numerical scoring system, or the actual number of vertigo episodes experienced in a given time period). 

###### 2.2.1. Global score

Thomsen 1981 reported on the change in vertigo using a patient‐rated score. This was recorded daily, and included the frequency, duration and severity of individual attacks with a score of 0 to 3 (0 = no attacks, 1 = weak attacks noted occasionally, 2 = strong attacks almost all the time but tolerable, 3 = severe attacks with interference with daily activities). The authors present the "median monthly score" for each participant, which is therefore presumably rated on a scale of 0 to 93 (maximum score 3 per day, up to 31 days per month). We are unsure whether this scoring system has been validated.

####### 2.2.1.1. At 3 to < 6 months

No data were reported at this time point.

####### 2.2.1.2. At 6 to ≤ 12 months

The mean change in vertigo at 12 months was ‐22.8 points for the endolymphatic sac decompression group, compared with ‐10.4 points for the sham surgery group (mean difference (MD) ‐12.39, 95% CI ‐22.81 to ‐1.97; 1 study; 29 participants; Analysis 2.2; very low‐certainty evidence).

####### 2.2.1.3. At > 12 months

No data were reported at this time point.

##### 2.3. Serious adverse events

No serious adverse events were reported ‐ it is unclear whether this was because no adverse events occurred, or simply because they were not assessed or reported.

##### 2.4. Disease‐specific health‐related quality of life

This outcome was not reported. 

##### 2.5. Change in hearing

Thomsen 1981 provided individual participant data for the hearing threshold at baseline and at the 12‐month and nine‐year follow‐up time point in graphical format. From these data we were able to calculate the change in hearing threshold.

###### 2.5.1. At 3 to < 6 months

No data were reported at this time point.

###### 2.5.2. At 6 to ≤ 12 months

The mean difference for those receiving endolymphatic sac surgery was ‐8.41 dB HL better (95% CI ‐17.00 to 0.18; 1 study; 30 participants; Analysis 2.3; very low‐certainty evidence). 

###### 2.5.3. At >12 months

The mean difference for those receiving endolymphatic sac surgery at nine years was 2.33 dB HL higher (i.e. worse, 95% CI ‐16.54 to 21.20; 1 study; 23 participants; Analysis 2.3; very low‐certainty evidence). 

##### 2.6. Change in tinnitus

This study did not assess tinnitus using a validated scale that considered the impact of tinnitus on quality of life. 

##### 2.7. Other adverse events

At the nine‐year follow‐up no adverse events were reported. However, seven patients were lost to follow‐up (six patients had died and one relocated).

## Discussion

### Ventilation tubes versus no intervention (plus medical therapy for both groups)

We identified a small amount of evidence for this comparison. Ventilation tubes may increase the proportion of people who have complete resolution of vertigo symptoms at two years, but the evidence was very uncertain. Similarly, there may be an increase in the proportion of people who have improvement in their hearing at two years, following ventilation tube insertion. However, we did not identify any evidence for our remaining outcomes (change in vertigo, disease‐specific health‐related quality of life, tinnitus, serious adverse events or other adverse effects). 

### Endolymphatic sac surgery versus sham surgery

All the evidence for this comparison was very uncertain, predominantly due to the very small study size (compounded by the high dropout rate over time) and wide confidence intervals for all effects seen. The proportion of people who experience an improvement in vertigo symptoms may be slightly higher after endolymphatic surgery at 12 months of follow‐up, but there was no difference at nine years of follow‐up. However, we are very uncertain about these effects. Similarly, the reduction in vertigo (as rated using a global score of vertigo severity) was greater (better) at 12 months for those who received endolymphatic sac surgery, but we are uncertain whether the size of this effect would be meaningful for people with Ménière's disease, and the confidence interval includes the possibility of a trivial difference. No data were available at longer‐term follow‐up for this outcome. 

At 12 months' follow‐up, those who received endolymphatic sac surgery had slightly better hearing, on average, when compared to those who received sham surgery, but the difference was small (mean difference of ‐8.41 dB HL), and the evidence was very uncertain. This effect was not seen at the nine‐year follow‐up point, when there was a trivial difference between the groups (mean difference of 2.33 dB HL). Again, all the evidence on hearing was of very low certainty, so we cannot be sure that these effects would be replicated in future studies. 

We did not identify any evidence on adverse effects of the intervention, disease‐specific health‐related quality of life or tinnitus. 

### Overall completeness and applicability of evidence

This review was conducted as part of a suite considering different interventions for Ménière's disease. A number of issues were identified as affecting the completeness and applicability of the evidence in all the reviews in this suite. These have been described in the companion review on 'Systemic pharmacological interventions for Ménière's disease' (Webster 2021a) and are replicated here, as they relate to this review:

- There is a paucity of evidence about all of these interventions, despite some of them being in common use for Ménière’s disease. All the evidence we found was of very low or low certainty, showing that we are unsure of the effects of the interventions, and future research may change the effect estimates a great deal. We did not find any evidence from randomised controlled trials for three of our planned comparison pairs (semi‐circular canal plugging/obliteration, vestibular nerve section or labyrinthectomy versus no treatment or placebo). 
- We were unable to carry out any meta‐analyses, as a single study contributed to each of our comparisons. Therefore, we were unable to pool data to achieve a more precise estimate of any effect. In other reviews in this suite, we noted that study authors often used different ways of measuring the same outcome, which also prevented data from being combined. For example, vertigo was assessed with either a global score, or a frequency score, which could not be combined, or hearing was assessed using a continuous scale or as an improvement above a certain threshold.
- Certain outcomes were only assessed by some included studies. Many studies did not assess the impact of the disease on quality of life or tinnitus at all. Potential adverse effects of the interventions were also often poorly reported or simply not assessed. Many surgical interventions have well‐recognised potential risks, such as infection, cerebrospinal fluid (CSF) leak or facial nerve injury. However, there was no description of these adverse effects in the included studies. We would expect to see a clear description of adverse events, even if this is simply a statement to acknowledge that no events occurred. 
- We noted that unvalidated rating scales were commonly used in the studies included, particularly when looking at the global impact of treatments for vertigo. When such scales are used, it is difficult to know if they are accurately assessing the outcome, and also what size of change on this scale represents a meaningful difference in the outcome (the minimally important difference).
- Finally, studies often failed to report clearly what treatments participants received before joining the trial, what maintenance treatment they continued on during the trial, and whether they received any additional treatments over the course of the trial. The impact of these additional treatments may be considerable, particularly for those studies with longer‐term follow‐up. Without knowing the background details of study participants (for example, the duration of their Ménière's disease, or what treatments they have tried in the past) it is difficult to identify the groups of people who may benefit from these treatments.

### Quality of the evidence

We used the GRADE approach to assess the certainty of the evidence in this review. The evidence identified was all low‐ or very low‐certainty, meaning that we are uncertain about the actual effect of these interventions for all of our outcomes. The main issues that affected the certainty of the evidence were the domains of study limitations, imprecision and 'other considerations' (i.e. publication bias). The different domains addressed by GRADE are considered in more detail below.

Both Kitahara 2016 and Thomsen 1981 had concerns noted regarding the potential for bias in the study design, conduct or reporting. Although Kitahara 2016 described adequate strategies to generate random assignments and to conceal allocation, it was an unblinded study. No attempts were made to mask participants, study personnel or outcome assessors to the interventions, leading to a high risk of performance and detection bias. Thomsen 1981 has been credited historically as the only blinded study involving surgery for Ménière’s disease. However, no attempts were made to describe the methods used in randomisation, leading to an unclear risk of selection bias. In addition, this study was at risk of attrition bias at the nine‐year follow‐up.

We have concerns about the risks of selective reporting for both studies. Kitahara 2016 described in their methods section that vertigo control would be assessed and reported as better/worse/unchanged (criteria for these categories were reported). However, only the number of participants in whom vertigo was completely resolved was reported. No published protocol could be found for Thomsen 1981 and it remains very uncertain if all of their recorded outcomes were fully reported. Further concerns were raised due to the re‐analysis of their study by Welling 2000, which led to different conclusions.

### Inconsistency

We conducted no meta‐analyses in the course of this review, therefore inconsistency did not impact on the certainty of the evidence.

### Indirectness

We rated down for indirectness if the majority of evidence for an outcome had come from a study where the population was not clearly defined (Thomsen 1981), or if there was significant concern over the methods used to measure an outcome (for example, use of an unvalidated scoring system for vertigo, as in Thomsen 1981). We rated down for indirectness if the outcome measured was different from that specified in our protocol (Webster 2021b). This was the case for the study Kitahara 2016, where the authors reported complete resolution of vertigo, rather than 'any improvement' in vertigo. 

### Imprecision

Both studies were small and, as discussed above, we were unable to carry out meta‐analyses. Therefore, the total sample size for each of our outcomes of interest was small, and reduced the certainty of the evidence.

For each analysis result, the width of the confidence interval is compared to the threshold for an important difference (details of how these thresholds were selected are described in the Methods section). If the confidence interval crosses this threshold ‐ and includes both the potential for an important benefit and the potential for a trivial effect, then the certainty of the evidence would be reduced by one level. If the confidence interval includes the possibility of *both* an important benefit and an important harm then the certainty would be reduced further. Therefore, it is important to agree on thresholds for this rating, i.e. where is the threshold, or cut‐point, between a trivial difference and a small, but important benefit or harm for each outcome? This question is difficult to answer, and requires input from people with balance disorders. As part of this review process, one of the author team (KW) joined some discussion groups for people with balance disorders, to try and obtain their views on quantifying an important and meaningful difference in treatment outcomes. However, the main theme that emerged from these discussions was that people were unable to give a specific threshold for each outcome. Instead, individuals tended to weigh up a variety of different factors when determining this threshold. The invasiveness and burden of taking the treatment would be taken into account, as well as potential side effects and the severity of their symptoms at that time. The GRADE working group would likely refer to this as a "fully contextualised approach", accounting for all aspects of the specific intervention in order to set thresholds for benefit (Zeng 2021). For this review we adopted a "minimally contextualised approach" and rated imprecision for each outcome according to specific, defined thresholds (as described in Methods). However, if the thresholds used are inappropriate then this may affect the certainty of the evidence (by a maximum of one level).

### Other considerations

We did not rate down the certainty of the evidence for other reasons. Publication bias is usually assessed as part of this domain. Although we are aware that this is an issue with many systematic reviews, we did not find strong indications of publication bias with this review.

### Potential biases in the review process

We planned to use the Cochrane Pregnancy and Childbirth Trustworthiness Tool to assess the included studies in this review. We had intended to exclude any study where there were concerns (as identified with this tool) from the main analyses. However, as described above, we were unable to determine whether the included studies would pass the screening tool ‐ either due to a lack of reporting in the original articles, or because we were unable to contact the authors to resolve any issues. If these studies were subsequently found to have genuine concerns over research integrity then this would further undermine our confidence in the findings of the review. However, as the evidence for these interventions is almost all very low‐certainty, we considered that this would not greatly impact the findings of the review. 

### Agreements and disagreements with other studies or reviews

An earlier Cochrane Review on this topic (Pullens 2013) included two trials ‐ the study Thomsen 1981, and one further trial by the same author that compared endolymphatic sac surgery to the insertion of ventilation tubes (Thomsen 1998). As there was no appropriate control arm in this study (both groups received a potentially active intervention) it was not included in our review. We identified two further reviews that also included only these two RCTs (Devantier 2019; Lim 2015). We also identified two reviews that included non‐randomised studies (Ballard 2019; Gronlund 2021), therefore cannot be easily compared with our own review. However, despite the slightly different approaches taken by other authors, we are in agreement with their general conclusions ‐ that there is a paucity of evidence for these interventions, and there is a need for high‐quality RCTs in this area. 

It should be noted that data from the study Thomsen 1981 were subsequently re‐analysed in an article by Welling 2000, and the authors came to different conclusions. The original publications concluded that there were no substantial differences between people who received endolymphatic sac decompression surgery and those who received sham surgery. The re‐analysis by Welling 2000 suggested that there may be a statistically significant difference between the two groups for certain outcomes, including postoperative dizziness, tinnitus (although this was not assessed with a validated scale) and a combined score of different symptoms. In keeping with Welling 2000, we used the raw data (extracted from graphs in the original publications) to conduct our analyses, and have likewise found a statistically significant difference between the groups in vertigo symptoms at 12 months. Nonetheless, the importance of this difference is difficult to judge ‐ as an unvalidated scoring system was used to assess vertigo ‐ and the certainty of the evidence remains very low, reflecting the uncertainty we have in the efficacy of this intervention.

## Authors' conclusions

**Implications for practice**

The current evidence base from randomised controlled trials (RCTs) for surgical interventions to treat Ménière's disease is very small. We identified only two studies, addressing different interventions (endolymphatic sac surgery and insertion of ventilation tubes). All evidence identified for our outcomes of interest was of very low‐ or low‐certainty, demonstrating that we have little confidence in the effect sizes seen, and further studies are very likely to change these estimates. We did not identify any evidence regarding potential adverse effects related to surgical interventions, although it is very likely that these interventions carry some degree of risk.

**Implications for research**

**Further research on surgical interventions for Ménière's disease**

The use of placebo controls in surgical trials has been carefully considered by the ASPIRE (Applying Surgical Placebo in Randomised Evaluations) group (Beard 2000). Colleagues in a number of different disciplines have highlighted that there should be greater use of these trials in situations where there is equipoise regarding the efficacy of surgery (Jonas 2015; Wartolowska 2014). There are several reasons why placebo‐controlled trials for surgery in Ménière's disease may be particularly important. 

- The subjective nature of many outcomes assessed in Ménière's disease (such as vertigo) means that they may be strongly influenced by the knowledge that people have of treatment they received. The only way to avoid this risk of bias is with the use of an appropriate placebo (sham surgery). 
- The mechanism of action for some surgical interventions used for Ménière's disease (such as endolymphatic sac surgery and ventilation tube insertion) is not well understood. This is due, in part, to limited understanding of the underlying disease process. Consequently the justification for carrying out these interventions is less clear and divides opinion. For destructive treatments such as labyrinthectomy or vestibular nerve section, the mechanism of action for eradication of vertigo is better understood. Therefore, despite the lack of RCTs assessing these interventions, there is also less uncertainty over their efficacy for improving vertigo symptoms.
- Some of the surgical interventions used for Ménière's disease have the potential for serious harms ‐ including cerebrospinal fluid leak and facial nerve palsy. It is difficult to justify these potential risks when the efficacy of the interventions is uncertain. Clear evidence of efficacy (or no effect) of these interventions, obtained from a placebo‐controlled trial, would enable people to weigh up the potential risks and benefits of the surgery. It is surprising that serious adverse events (or other adverse effects) were not reported by the authors of the trials included in this review. Some complications might be anticipated even when surgery is fairly minor (such as insertion of ventilation tubes). When considering undertaking a surgical procedure of unknown benefit, the potential risks of surgery are of great importance. Therefore, we would strongly advocate that authors of future trials ensure that data on adverse effects of these interventions are systematically collected and fully reported. 
- Finally, the disease fluctuates over time, therefore assessment of efficacy using 'before‐and‐after' study designs may be ineffective. Participants in a trial of a surgical intervention are likely to be experiencing severe symptoms at the time of enrolment, and it may be anticipated that these symptoms will improve over time, even if no surgery is conducted. This phenomenon was noted by Kerr 1998, who assessed a cohort of 23 people awaiting surgery for Ménière's disease. Twelve of these experienced an improvement in their symptoms within six to eight weeks of agreeing to surgery, and therefore did not require an operation. 

Given that surgical interventions for Ménière's disease are relatively rare, future studies are likely to require multicentre collaboration. There may also need to be clear consensus from stakeholders (especially people with Ménière's disease) to prioritise which are the most important surgical interventions to assess. This may include the most commonly performed surgical intervention, or an intervention of uncertain benefit where the risk of harm is the highest. 

**Further research on other interventions for Ménière's disease**

This review was conducted as part of a suite regarding a number of different interventions for Ménière's disease. Many of the conclusions below are relevant to all of these reviews and are replicated across the suite.

The lack of high‐certainty, RCT evidence suggests that well‐conducted studies with larger numbers of participants are required to appropriately assess the efficacy (and potential harms) of these interventions. However, there also needs to be more clarity on which outcomes studies should assess, and when and how to assess them. Vertigo is a notoriously difficult symptom to assess, and there is great variety in the methods used to record and report this symptom in the studies we have identified. 

There is a clear need for consensus on which outcomes are important to people with Ménière’s disease, so that future studies can be designed with this in mind. Development of a core outcome set would be preferable as a guide for future trials. We understand that development of a core outcome set for Ménière's disease was underway, with a project registered on the COMET website (https://www.comet-initiative.org/Studies/Details/818), but we have been unable to identify any results of this project, or ascertain whether it is ongoing. If a core outcome set is developed, this should include details on the recommended methods used to measure outcomes, ensuring that these are validated, reliable tools. Monitoring and reporting of adverse effects should be considered a routine part of any study, and should always occur ‐ this is inconsistent at present. Agreement is also needed on the appropriate times at which outcomes should be measured to adequately assess the different interventions.

Any decisions about which outcomes to measure, how to measure them and when to measure them must be made with input from people with Ménière’s disease, to ensure that the outcomes reported by trialists (and future systematic reviews) are relevant to those with the disease. 

For those considering development of a core outcome set, we would highlight that the use of the dichotomous outcome 'improvement' or 'no improvement' of vertigo may cause difficulties when interpreting the results. Individuals with Ménière's disease typically experience fluctuations in disease severity over time. Furthermore, they may have enrolled in a clinical trial at a time when their symptoms were severe. Therefore there is likely to be a natural tendency to improve over time, even for those who do not receive an intervention. The high rate of improvement in those who receive no treatment means that smaller studies are likely to be underpowered to detect a true effect of treatment. Ideally, agreement should be reached on what constitutes a *meaningful improvement* in vertigo symptoms, rather than simply considering any improvement as a positive outcome. 

Trialists should also be clear about the treatments that participants received before entry to the trial, throughout the trial, and the need for additional treatment during the course of the trial. People with Ménière's disease need to be able to understand whether interventions work in all people with the disease, or whether they might work best during certain phases of the disease ‐ perhaps as a first‐line therapy, or for people in whom other treatments have failed. 

## History

Protocol first published: Issue 12, 2021

## Appendices

### Appendix 1. AAO‐HNS definition of Ménière's disease

Definite Ménière's disease:

- Two or more spontaneous episodes of vertigo, each lasting 20 minutes to 12 hours.
- Audiometrically documented low‐ to medium‐frequency sensorineural hearing loss in one ear, defining the affected ear on at least one occasion before, during or after one of the episodes of vertigo.
- Fluctuating aural symptoms (hearing, tinnitus or fullness) in the affected ear.
- Not better accounted for by another vestibular diagnosis.

Probable Ménière's disease: 

- Two or more spontaneous episodes of vertigo, each lasting 20 minutes to 24 hours.
- Fluctuating aural symptoms (hearing, tinnitus or fullness) in the affected ear.
- Not better accounted for by another vestibular diagnosis.

Taken from Lopez‐Escamez 2015.

### Appendix 2. Search strategy

This search strategy has been designed to identify all relevant studies for a suite of reviews on various interventions for Ménière's disease. 

**Table:**

**CENTRAL (CRS) **	**Cochrane ENT Register (CRS) **	**MEDLINE (Ovid) **
1 MESH DESCRIPTOR Endolymphatic Hydrops EXPLODE ALL AND CENTRAL:TARGET 2 meniere*):AB,EH,KW,KY,MC,MH,TI,TO AND CENTRAL:TARGET 3 (endolymphatic near hydrops):AB,EH,KW,KY,MC,MH,TI,TO AND CENTRAL:TARGET 4 (labyrinth* near hydrops):AB,EH,KW,KY,MC,MH,TI,TO AND CENTRAL:TARGET 5 (labyrinth* near syndrome):AB,EH,KW,KY,MC,MH,TI,TO AND CENTRAL:TARGET 6 (aural near vertigo):AB,EH,KW,KY,MC,MH,TI,TO AND CENTRAL:TARGET 7 (labyrinth* near vertigo):AB,EH,KW,KY,MC,MH,TI,TO AND CENTRAL:TARGET 8 (cochlea near hydrops):AB,EH,KW,KY,MC,MH,TI,TO AND CENTRAL:TARGET 9 (vestibular near hydrops):AB,EH,KW,KY,MC,MH,TI,TO AND CENTRAL:TARGET 10 #1 OR #2 OR #3 OR #4 OR #5 OR #6 OR #7 OR #8 OR #9 AND CENTRAL:TARGET	1 MESH DESCRIPTOR Endolymphatic Hydrops EXPLODE ALL AND INREGISTER 2 (meniere*):AB,EH,KW,KY,MC,MH,TI,TO AND INREGISTER 3 (endolymphatic near hydrops):AB,EH,KW,KY,MC,MH,TI,TO AND INREGISTER 4 (labyrinth* near hydrops):AB,EH,KW,KY,MC,MH,TI,TO AND INREGISTER 5 (labyrinth* near syndrome):AB,EH,KW,KY,MC,MH,TI,TO AND INREGISTER 6 (aural near vertigo):AB,EH,KW,KY,MC,MH,TI,TO AND INREGISTER 7 (labyrinth* near vertigo):AB,EH,KW,KY,MC,MH,TI,TO AND INREGISTER 8 (cochlea near hydrops):AB,EH,KW,KY,MC,MH,TI,TO AND INREGISTER 9 (vestibular near hydrops):AB,EH,KW,KY,MC,MH,TI,TO AND INREGISTER 10 #1 OR #2 OR #3 OR #4 OR #5 OR #6 OR #7 OR #8 OR #9 AND INREGISTER 11 INREGISTER 12 * AND CENTRAL:TARGET 13 #11 NOT #12 14 #10 AND #13	1 exp Endolymphatic Hydrops/ 2 meniere*.ab,ti. 3 (endolymphatic adj3 hydrops).ab,ti. 4 (labyrinth* adj3 hydrops).ab,ti. 5 (labyrinth* adj3 syndrome).ab,ti. 6 (aural adj3 vertigo).ab,ti. 7 (labyrinth* adj3 vertigo).ab,ti. 8 (cochlea adj3 hydrops).ab,ti. 9 (vestibular adj3 hydrops).ab,ti. 10 1 or 2 or 3 or 4 or 5 or 6 or 7 or 8 or 9 11 randomized controlled trial.pt. 12 controlled clinical trial.pt. 13 randomized.ab. 14 placebo.ab. 15 drug therapy.fs. 16 randomly.ab. 17 trial.ab. 18 groups.ab. 19 11 or 12 or 13 or 14 or 15 or 16 or 17 or 18 20 exp animals/ not humans.sh. 21 19 not 20 22 10 and 21
**Embase (Ovid) **	**Web of Science Core Collection (Web of Knowledge) **	**Trial Registries **
1 exp Meniere disease/ 2 meniere*.ab,ti. 3 (endolymphatic adj3 hydrops).ab,ti. 4 (labyrinth* adj3 hydrops).ab,ti. 5 (labyrinth* adj3 syndrome).ab,ti. 6 (aural adj3 vertigo).ab,ti. 7 (labyrinth* adj3 vertigo).ab,ti. 8 (cochlea adj3 hydrops).ab,ti. 9 (vestibular adj3 hydrops).ab,ti. 10 1 or 2 or 3 or 4 or 5 or 6 or 7 or 8 or 9 11 Randomized controlled trial/ 12 Controlled clinical study/ 13 Random$.ti,ab. 14 randomization/ 15 intermethod comparison/ 16 placebo.ti,ab. 17 (compare or compared or comparison).ti. 18 ((evaluated or evaluate or evaluating or assessed or assess) and (compare or compared or comparing or comparison)).ab. 19 (open adj label).ti,ab. 20 ((double or single or doubly or singly) adj (blind or blinded or blindly)).ti,ab. 21 double blind procedure/ 22 parallel group$1.ti,ab. 23 (crossover or cross over).ti,ab. 24 ((assign$ or match or matched or allocation) adj5 (alternate or group$1 or intervention$1 or patient$1 or subject$1 or participant$1)).ti,ab. 25 (assigned or allocated).ti,ab. 26 (controlled adj7 (study or design or trial)).ti,ab. 27 (volunteer or volunteers).ti,ab. 28 human experiment/ 29 trial.ti. 30 11 or 12 or 13 or 14 or 15 or 16 or 17 or 18 or 19 or 20 or 21 or 22 or 23 or 24 or 25 or 26 or 27 or 28 or 29 31 (random$ adj sampl$ adj7 ("cross section$" or questionnaire$1 or survey$ or database$1)).ti,ab. 32 comparative study/ or controlled study/ 33 randomi?ed controlled.ti,ab. 34 randomly assigned.ti,ab. 35 32 or 33 or 34 36 31 not 35 37 Cross‐sectional study/ 38 randomized controlled trial/ or controlled clinical study/ or controlled study/ 39 (randomi?ed controlled or control group$1).ti,ab. 40 38 or 39 41 37 not 40 42 (((case adj control$) and random$) not randomi?ed controlled).ti,ab. 43 (Systematic review not (trial or study)).ti. 44 (nonrandom$ not random$).ti,ab. 45 Random field$.ti,ab. 46 (random cluster adj3 sampl$).ti,ab. 47 (review.ab. and review.pt.) not trial.ti. 48 we searched.ab. 49 review.ti. or review.pt. 50 48 and 49 51 update review.ab. 52 (databases adj4 searched).ab. 53 (rat or rats or mouse or mice or swine or porcine or murine or sheep or lambs or pigs or piglets or rabbit or rabbits or cat or cats or dog or dogs or cattle or bovine or monkey or monkeys or trout or marmoset$1).ti. and animal experiment/ 54 36 or 41 or 42 or 43 or 44 or 45 or 46 or 47 or 50 or 51 or 52 55 30 not 54 56 10 and 55	# 12 #11 AND #10 Indexes=SCI‐EXPANDED, SSCI, A&HCI, CPCI‐S, CPCI‐SSH, BKCI‐S, BKCI‐SSH, ESCI, CCR‐EXPANDED, IC Timespan=All years # 11 TOPIC: ((randomised OR randomized OR randomisation OR randomisation OR placebo* OR (random* AND (allocat* OR assign*) ) OR (blind* AND (single OR double OR treble OR triple) ))) Indexes=SCI‐EXPANDED, SSCI, A&HCI, CPCI‐S, CPCI‐SSH, BKCI‐S, BKCI‐SSH, ESCI, CCR‐EXPANDED, IC Timespan=All years # 10 #9 OR #8 OR #7 OR #6 OR #5 OR #4 OR #3 OR #2 OR #1 Indexes=SCI‐EXPANDED, SSCI, A&HCI, CPCI‐S, CPCI‐SSH, BKCI‐S, BKCI‐SSH, ESCI, CCR‐EXPANDED, IC Timespan=All years # 9 TOPIC: (vestibular NEAR/3 hydrops) Indexes=SCI‐EXPANDED, SSCI, A&HCI, CPCI‐S, CPCI‐SSH, BKCI‐S, BKCI‐SSH, ESCI, CCR‐EXPANDED, IC Timespan=All years # 8 TOPIC: (cochlea NEAR/3 hydrops) Indexes=SCI‐EXPANDED, SSCI, A&HCI, CPCI‐S, CPCI‐SSH, BKCI‐S, BKCI‐SSH, ESCI, CCR‐EXPANDED, IC Timespan=All years # 7 TOPIC: (labyrinth* NEAR/3 vertigo) Indexes=SCI‐EXPANDED, SSCI, A&HCI, CPCI‐S, CPCI‐SSH, BKCI‐S, BKCI‐SSH, ESCI, CCR‐EXPANDED, IC Timespan=All years # 6 TOPIC: (labyrinth* adj3 vertigo) Indexes=SCI‐EXPANDED, SSCI, A&HCI, CPCI‐S, CPCI‐SSH, BKCI‐S, BKCI‐SSH, ESCI, CCR‐EXPANDED, IC Timespan=All years # 5 TOPIC: (aural NEAR/3 vertigo) Indexes=SCI‐EXPANDED, SSCI, A&HCI, CPCI‐S, CPCI‐SSH, BKCI‐S, BKCI‐SSH, ESCI, CCR‐EXPANDED, IC Timespan=All years # 4 TOPIC: (labyrinth* NEAR/3 syndrome) Indexes=SCI‐EXPANDED, SSCI, A&HCI, CPCI‐S, CPCI‐SSH, BKCI‐S, BKCI‐SSH, ESCI, CCR‐EXPANDED, IC Timespan=All years # 3 TOPIC: (labyrinth* NEAR/3 hydrops) Indexes=SCI‐EXPANDED, SSCI, A&HCI, CPCI‐S, CPCI‐SSH, BKCI‐S, BKCI‐SSH, ESCI, CCR‐EXPANDED, IC Timespan=All years # 2 TOPIC: (endolymphatic NEAR/3 hydrops) Indexes=SCI‐EXPANDED, SSCI, A&HCI, CPCI‐S, CPCI‐SSH, BKCI‐S, BKCI‐SSH, ESCI, CCR‐EXPANDED, IC Timespan=All years # 1 TOPIC: (meniere*) Indexes=SCI‐EXPANDED, SSCI, A&HCI, CPCI‐S, CPCI‐SSH, BKCI‐S, BKCI‐SSH, ESCI, CCR‐EXPANDED, IC Timespan=All years	Clinicaltrials.gov menieres or meniere or meniere's | Interventional Studies ICTRP Meniere*

The date restrictions applied to the September 2022 update searches were as follows:

**Table:**

** **	**September 2022 update**
**CENTRAL**	15/09/2021_TO_14/09/2022:CRSINCENTRAL AND CENTRAL:TARGET
**ENT register**	No new records added to register since search was run
**MEDLINE**	23 limit 22 to ed=20210915‐20220914 24 limit 22 to dt=20210915‐20220914 25 23 or 24
**Embase**	57 limit 56 to dd=20210915‐20220914
**Web of Science**	Timespan: 2021‐09‐15 to 2022‐09‐14 (Index Date)
**Clinicaltrials.gov**	First posted from 09/15/2021 to 09/14/2022
**ICTRP**	Date of registration after 15/09/2021
**Google Scholar**	Year: 2021 or 2022

### Appendix 3. Trustworthiness Screening Tool

This screening tool has been developed by Cochrane Pregnancy and Childbirth. It includes a set of predefined criteria to select studies that, based on available information, are deemed to be sufficiently trustworthy to be included in the analysis. These criteria are:

**Research governance**

- Are there any retraction notices or expressions of concern listed on the Retraction Watch Database relating to this study?
- Was the study prospectively registered (for those studies published after 2010)? If not, was there a plausible reason?
- When requested, did the trial authors provide/share the protocol and/or ethics approval letter?
- Did the trial authors engage in communication with the Cochrane Review authors within the agreed timelines?
- Did the trial authors provide IPD data upon request? If not, was there a plausible reason?

**Baseline characteristics**

- Is the study free from characteristics of the study participants that appear too similar (e.g. distribution of the mean (SD) excessively narrow or excessively wide, as noted by Carlisle 2017)?

**Feasibility**

- Is the study free from characteristics that could be implausible? (e.g. large numbers of women with a rare condition (such as severe cholestasis in pregnancy) recruited within 12 months);
- In cases with (close to) zero losses to follow‐up, is there a plausible explanation?

**Results**

- Is the study free from results that could be implausible? (e.g. massive risk reduction for main outcomes with small sample size)?
- Do the numbers randomised to each group suggest that adequate randomisation methods were used (e.g. is the study free from issues such as unexpectedly even numbers of women 'randomised’ including a mismatch between the numbers and the methods, if the authors say 'no blocking was used’ but still end up with equal numbers, or if the authors say they used 'blocks of 4’ but the final numbers differ by 6)?

Studies assessed as being potentially 'high risk’ will be not be included in the review. Where a study is classified as 'high risk’ for one or more of the above criteria we will attempt to contact the study authors to address any possible lack of information/concerns. If adequate information remains unavailable, the study will remain in 'awaiting classification’ and the reasons and communications with the author (or lack of) described in detail.

The process is described in full in Figure. 

## Data and analyses

**Comparison 1:** Ventilation tubes versus no treatment

Outcome or subgroup title	No. of studies	No. of participants	Statistical method	Effect size
1.1 Improvement in frequency of vertigo (complete resolution) at > 12 months	1	133	Risk Ratio (M‐H, Fixed, 95% CI)	1.55 [1.22, 1.97]
1.2 Change in hearing: improvement in hearing at > 12 months	1	133	Risk Ratio (M‐H, Fixed, 95% CI)	4.89 [1.97, 12.14]

**Comparison 2:** Endolymphatic sac decompression versus placebo (sham surgery)

Outcome or subgroup title	No. of studies	No. of participants	Statistical method	Effect size
2.1 Improvement in vertigo (complete resolution)	1		Risk Ratio (M‐H, Random, 95% CI)	Subtotals only
2.1.1 6 to ≤ 12 months	1	30	Risk Ratio (M‐H, Random, 95% CI)	1.30 [0.86, 1.96]
2.1.2 > 12 months	1	23	Risk Ratio (M‐H, Random, 95% CI)	0.91 [0.72, 1.16]
2.2 Change in vertigo	1	29	Mean Difference (IV, Random, 95% CI)	‐12.39 [‐22.81, ‐1.97]
2.2.1 At 6 to ≤ 12 months	1	29	Mean Difference (IV, Random, 95% CI)	‐12.39 [‐22.81, ‐1.97]
2.3 Change in hearing	1		Mean Difference (IV, Random, 95% CI)	Subtotals only
2.3.1 6 to ≤ 12 months	1	30	Mean Difference (IV, Random, 95% CI)	‐8.41 [‐17.00, 0.18]
2.3.2 > 12 months	1	23	Mean Difference (IV, Random, 95% CI)	2.33 [‐16.54, 21.20]

## Characteristics of studies

### Characteristics of included studies [ordered by study ID]

**Table:** Kitahara 2016

***Study characteristics***		
Methods	Four‐arm, parallel‐group, open‐label RCT with 24 months total duration of treatment and follow‐up Two arms are relevant to this review and the data below pertain to these arms. The 3rd and 4th arms (abundant water intake and sleeping in darkness) are relevant for, and included in, a separate review in this suite ('Lifestyle and dietary interventions for Ménière's disease'; Webster 2022).	
Participants	**Setting: **single‐centre study. Recruitment took place in the vertigo and dizziness department of Osaka Rosai Hospital in Japan from January 2010 until December 2013. **Sample size:** **Number randomised:** 148 participants included in the 2 arms relevant to this review (297 total in the trial) **Number completed:** 133 participants included in the 2 arms relevant to this review (263 total in the trial) **Participant baseline characteristics** **Age:** Ventilation tubes group: mean 51.7 years (SD 14.7) Control group: mean 49.7 years (SD 16.5) **Gender:** Ventilation tubes group: 22 males (34.9%): 41 females (65.1%) Control group: 25 males (35.7%): 45 females (64.3%) **Probable/definite Ménière's disease: ** All definite disease **Duration of disease: ** Ventilation tubes group: mean 37.2 months (SD 20.9) Control group: mean 30.3 months (SD 22.7) **Attack frequency at baseline: ** Ventilation tubes group: mean 1.8 attacks per month (SD 1.1) Control group: mean 1.6 attacks per month (SD 1) **Hearing loss at baseline:** Ventilation tubes group: mean 49.5 dB hearing level (SD 13.2) Control group: mean 47.3 dB hearing level (SD 13.4) **Measure of tinnitus at baseline: ** Not reported **Number of participants with bilateral disease: ** None (table of demographics reports laterality of disease for each participant; no participant is listed as having bilateral disease, therefore we assume all were unilateral) **Inclusion criteria:** definite Ménière's disease according to the AAO‐HNS 1995 criteria. Age ≥ 20 years. Eligible patients were those in whom 3 to 6 months of "basically fixed forms of medical treatment" had produced insufficient benefit (i.e. recurrent vertigo attacks and/or no improvement in sensorineural hearing loss) and for whom surgical treatment would otherwise be considered. Medical treatments included diuretics, betahistine, diphenidol, dimenhydrinate and diazepam. No further information on doses or treatments in each group was provided. **Exclusion criteria:** cardiac disease; renal dysfunction; middle ear problems; nyctophobia (those who cannot sleep in darkness). Participants were excluded from the trial if they did not adhere to their treatment (< 75% adherence). **Criteria for diagnosis of M**é**ni**è**re's disease:** definite Ménière's disease according to the AAO‐HNS 1995 criteria	
Interventions	**Intervention (n = 74 randomised, n = 63 completed):** ventilation tubes plus medication; they also continued traditional medication as per the control group **Comparator (n = 74 randomised, n = 70 completed):** medication only; participants continued to receive traditional oral medications: "Medical treatments were fixed basically including diuretics, betahistine, diphenidol, dimenhydrinate, and diazepam, all considered effective for persistent symptoms of Meniere’s disease" **Background interventions administered to all participants:** as described above, both groups received oral medication	
Outcomes	**Primary outcomes relevant to this review:** **Improvement in vertigo** Improvement of vertigo was assessed using the control of vertigo scale, according to the AAO‐HNS 1995, with a score of < 0.8 being regarded as improvement. However, the article only reports the proportion of participants who had complete resolution of vertigo. **Change in vertigo** Not reported **Serious adverse events** Not reported; trial registration document does list "all cause mortality" as the primary outcome measure **Secondary outcomes relevant to this review:** **Disease‐specific health‐related quality of life** Not assessed or reported **Hearing** Assessed with pure tone audiometry with an average of four frequencies (0.5 kHz, 1 kHz, 2 kHz and 4 kHz). Reported as a dichotomous outcome: improved/not improved, with a threshold of a decrease of 10 dB representing improvement. **Tinnitus ** Not assessed or reported **Other adverse effects** Not assessed or reported **Other outcomes reported in the study:** Blood results for stress (pAVP and cortisol) Questionnaires measuring psychological factors (SDS and SRS‐18)	
Funding sources	"This study was supported in part by JSPS KAKENHI Grant (2016‐2018) and by Grant‐in‐Aid for Research on Intractable Vestibular Disorder from Japan AMED (2015‐2017). The funders above had no role in study design, data collection and analysis, decision to publish, or preparation of the manuscript."	
Declarations of interest	The authors have declared that no competing interests exist	
Notes	**Research integrity checklist:** No retractions/expressions of concern were identified Baseline characteristics of the groups were not excessively similar Dropouts and withdrawals were described The effect size for all of the interventions is considerably larger than for the control group, despite all participants in the trial receiving standard treatment for Ménière's disease, and the control group achieving considerable improvement over the course of the trial Blocked randomisation was used, accounting for similar numbers of participants allocated to each group	
***Risk of bias***		
**Bias**	**Authors' judgement**	**Support for judgement**
Random sequence generation (selection bias)	Low risk	Quote: "The remaining 297 definite Meniere's patients were randomly assigned through computer‐generated block randomization organized by the clinical study section at Osaka Rosai Hospital to one of four treatment groups." Comment: computer‐generated block randomisation by a third party.
Allocation concealment (selection bias)	Low risk	Quote: "The remaining 297 definite Meniere's patients were randomly assigned through computer‐generated block randomization organized by the clinical study section at Osaka Rosai Hospital to one of four treatment groups." Comment: computer‐generated block randomisation and allocation by a third party.
Blinding of participants and personnel (performance bias) All outcomes	High risk	Comment: this was an open‐label study. Participants and study personnel were aware of group allocation.
Blinding of outcome assessment (detection bias) All outcomes	High risk	Comment: this was an open‐label study. Participants were aware of their group allocation, and outcomes are reported by (unblinded) participants and study personnel.
Incomplete outcome data (attrition bias) All outcomes	Low risk	Comment: the number of dropouts was low (< 10%) and balanced across the groups. This should have been insufficient to cause a significant change in the estimated effect size.
Selective reporting (reporting bias)	High risk	Quote: "A ratio of pre/post‐treatment values of ≤0.8 was regarded as "better", or ≥1.2 as "worse" and the remainder as no change" Comment: vertigo improvement was stated to be assessed as better/worse/unchanged in the methods. However, the authors only report the number of participants in whom vertigo resolved completely over the course of the study. Although the study was prospectively registered with ClinicalTrials.gov, the only outcome listed here is "all cause mortality". This seems a surprising choice of primary outcome measure, and is also not reported in the publication.
Other bias	Low risk	Comment: no other concerns were identified.

**Table:** Thomsen 1981

***Study characteristics***		
Methods	Two‐arm, parallel‐group, double‐blind randomised controlled trial with 12 months of follow‐up. Additional follow‐up continued for up to 9 years.	
Participants	**Setting: **participants were recruited from 2 university ENT departments in Copenhagen, Denmark. The study dates are not reported. **Sample size:** **Number randomised:** 30 participants **Number completed:** 30 participants (at 12 months) **Participant baseline characteristics** **Age: ** Endolymphatic sac decompression group: 49.9 years (range 25 to 63 years) Control (mastoidectomy) group: 53.9 years (range 28 to 69 years) **Gender: ** Endolymphatic sac decompression group: 9 males (60%): 6 females (40%) Control (mastoidectomy) group: 9 males (60%): 6 females (40%) **Probable/definite Ménière's disease:** not reported **Duration of disease:** not reported **Attack frequency at baseline:** not reported **Hearing loss at baseline (data estimated from figure):** Endolymphatic sac decompression group: mean pre‐operative hearing level 55.28 dB (SD 10.89) Control (mastoidectomy) group: mean pre‐operative hearing level 50.28 dB (SD 15.84) **Measure of tinnitus at baseline:** not reported **Number of participants with bilateral disease: ** Endolymphatic sac decompression group: 2/15 Control (mastoidectomy) group: 3/15 **Inclusion criteria:** presence of typical attacks of fluctuating hearing loss, tinnitus and vertigo. Attacks often accompanied by nausea, vomiting and pressure in the ear. At least 1 attack every 2 weeks. A history of Ménière's disease of not less than 6 months, but not longer than 5 years. Normal renal, cardiac and thyroid function. Considered 'psychologically normal'. Able to participate in the study for 12 months. **Exclusion criteria:** tumours of the cerebellopontine angle; allergies **Diagnosis of Ménière's disease:** no criteria stated. Description in the text states: "Presence of typical attacks of fluctuating hearing loss, tinnitus and vertigo: often accompanied by nausea, vomiting and pressure in the ear".	
Interventions	**Intervention (n = 15 randomised, n = 15 completed at 12 months):** regular endolymphatic sac shunt operation with insertion of Silastic into the sac, draining out into the mastoid cavity **Comparator (n = randomised, n = completed):** regular mastoidectomy, "at which much care was taken not to remove the bone over the endolymphatic sac, in order to avoid a decompression." **Background interventions administered to all participants:** all participants had "regular medical treatments initiated by their own otologists" for 3 months prior to their surgery. No further information on what this treatment was.	
Outcomes	**Primary outcomes relevant to this review:** **Improvement in vertigo** Reported according to the AAOO 1972 criteria for improvement. This considers vertigo and hearing, but classifies improvement in vertigo as only complete resolution or continued vertigo. **Change in vertigo** Participants rated their dizziness and vertigo daily with a scale of 0 to 3: 0 = no dizziness/vertigo; 1 ‐ weak‐rare, noted occasionally; 2 = strong‐significant, almost all of the time but tolerable; 3 = severe, interferes with daily activities, patient stays in bed. Data were presented graphically in the article. **Serious adverse events** Not reported **Secondary outcomes relevant to this review:** **Disease‐specific health‐related quality of life** Not reported **Hearing** Assessed with pure tone audiometry (average values at 250 Hz, 500 Hz and 1000 Hz). Data are reported graphically, however there is a discrepancy in the results presented in different articles. Therefore we have only included the data reported at 12 months in the review. **Tinnitus ** Not assessed with a validated questionnaire **Other adverse effects** Not reported **Other outcomes reported in the study:** Pressure in the ear, assessed with 3‐point daily scale Nausea and vomiting, assessed with 3‐point daily scale Total score of disease severity, incorporating vertigo/dizziness, nausea and vomiting, tinnitus, hearing loss, pressure in ears) Investigator global score of disease severity (range 0 to 10) Investigator assessment of benefit of surgery (very beneficial, fairly beneficial and no effect) Patient's evaluation of the value of the operation	
Funding sources	No funding is declared	
Declarations of interest	No declaration is made	
Notes	**Research integrity checklist:** No retractions or expressions of concern were identified Trial registration was not applicable (the study was published in 1981) Baseline characteristics of the 2 groups were not reported in full, therefore unable to assess fully. Gender of participants was identical in each group. No participants were reported to have dropped out during the first 12 months of the trial. Some dropout was reported over the subsequent 9‐year follow‐up. No implausible results are reported Block randomisation is not described, although equal numbers were assigned to each group. Furthermore, stratified randomisation was not described despite equal numbers of men and women in each group.	
***Risk of bias***		
**Bias**	**Authors' judgement**	**Support for judgement**
Random sequence generation (selection bias)	Unclear risk	Quote: "The patients were assigned randomly for each treatment group". Comment: method of randomisation is not described.
Allocation concealment (selection bias)	Unclear risk	Quote: "The patients were assigned randomly for each treatment group". Comment: methods used to conceal the group allocation were not described.
Blinding of participants and personnel (performance bias) All outcomes	Low risk	Quote: "the patients operated on at the Rigshospital were controlled every month at Gentofte hospital and vice versa in order to deprive the investigator of any information about the operative procedure employed. A standardized operation description was written in the patients’ charts in order to deprive the nursing staff at the hospital of information on the character of the operation". In subsequent iterations of this study (3‐year follow up): "the patients are still unaware of which kind of surgery was performed, and the investigator who had controlled the patients and made the interviews still does not know which groups the patients belong to." Comment: authors describe that participants were unaware of the procedure that they underwent. Adequate efforts to ensure that study personnel remained blinded to the treatment allocation.
Blinding of outcome assessment (detection bias) All outcomes	Low risk	Comment: primary outcomes based on vertigo diary completed by blinded participants. Follow‐up for patients was provided by a separate physician who was reported to be blinded to the nature of the operation.
Incomplete outcome data (attrition bias) All outcomes	High risk	Comment: considerable loss to follow‐up by 9 years (4/15 in the active group (26%) and 3/15 in the placebo group (20%) had dropped out). Full follow‐up until 12 months, therefore attrition bias is not a concern at the earlier time points.
Selective reporting (reporting bias)	Unclear risk	Comment: no published protocol. Unclear whether all recorded outcomes are fully reported. No details on adverse effects, which is surprising for a trial of a surgical intervention.
Other bias	High risk	Comment: some concern over data presented due to subsequent re‐analysis of the data in this trial leading to different conclusions (see Welling 2000). There was also no control around how patients were treated medically during the 9‐year follow‐up period. With such low numbers this could have had a significant impact on the results.

pAVP: stress hormone vasopressin; SD: standard deviation; SDS: Self‐rating Depression Scale; SRS‐18: Stress Response Scale‐18; RCT: randomised controlled trial

### Characteristics of excluded studies [ordered by study ID]

**Table:**

Study	Reason for exclusion
Arenberg 1981	This is a critique of the study Thomsen 1981, with no primary data
Arslan 1971	This is a description of a surgical technique, not an RCT
Beck 1985	This is a 'roundtable' discussion/review article, not an RCT
Bojrab 2018	This RCT compares 2 different surgical techniques for endolymphatic sac decompression (with and without intra‐sac injections of dexamethasone), therefore the comparator is incorrect for this review
Conde Jahn 1965	This is a narrative review article, not an RCT
Heatley 1991	This is a retrospective study comparing individuals who received endolymphatic sac decompression with those who received medical management, not an RCT
Kitahara 2008	This RCT compared endolymphatic sac drainage plus instillation of steroids to endolymphatic sac drainage alone. Although a comparator arm of no treatment was included, this comprised participants who declined surgery, not people who were randomised to the group.
Lu 2002	This study compared 2 different surgical techniques, therefore the comparator is incorrect, and it was also not an RCT
NCT01574313	The comparator for this RCT is not correct: stellate ganglion block alone is compared to medical management
NCT04847700	This ongoing study compares 2 different surgical techniques, therefore there is no relevant comparator for this review
Richards 1971	This is a narrative review article, not an RCT
Sakagami 2015	This study assigned participants to receive endolymphatic sac drainage with or without steroid instillation, therefore the comparator is not appropriate for this review. Participants were not randomised to each group.
Saliba 2015	This RCT compared endolymphatic duct blockage to endolymphatic sac decompression, therefore the comparator is not relevant for this review
Schenck 2021	This RCT will compare endolymphatic sac decompression with endolymphatic duct blockage; the comparator is not appropriate for this review
Thomsen 1998	This RCT compared insertion of ventilation tubes with insertion of an endolymphatic sac shunt; the comparator is inappropriate for this review
UMIN000005544	This is a prospective cohort study, not an RCT
Welling 1996	This is a prospective cohort study, with participants matched to historical controls, not an RCT
Welling 2000	This is a re‐analysis of the data from Thomsen 1981, which does not include any primary data
Xu 2022	All participants in this study underwent surgery. The only difference between the groups was that one group also received installation of local steroids at the time of surgery.

RCT: randomised controlled trial

### Characteristics of ongoing studies [ordered by study ID]

**Table:** NCT04835688

Study name	Ventilation tube insertion for unilateral Ménière's disease
Methods	Quadruple‐blind randomised controlled trial, to be conducted in Denmark
Participants	Estimated enrolment: 104 participants Inclusion criteria: Aged 18 years or older Patients with definite or probable unilateral Ménière's disease according to the diagnostic criteria formulated by the Classification Committee of the Bárány Society, The Japan Society for Equilibrium Research, the European Academy of Otology and Neurotology (EAONO), the Equilibrium Committee of the American Academy of Otolaryngology ‐ Head and Neck Surgery (AAO‐HNS), and the Korean Balance Society Exclusion criteria: Bilateral Ménière's disease Additional neurotological disorders (e.g. vestibular migraine, vertebrobasilar transient ischaemic attack or acoustic neuroma) Previous surgical therapy such as intratympanic gentamicin or endolymphatic sac surgery Expected problems with adherence to the study protocol (dementia, non‐fluent in Danish, substance abuse, etc.) Previous treatment with transmyringeal ventilation tubes after childhood A serious illness that might interfere with treatment or follow‐up
Interventions	Intervention: ventilation tube insertion The tympanic membrane will be anaesthetised. An incision will be made, usually in the lower, anterior quadrant of the tympanic membrane and the transmyringeal ventilation tube is inserted. Comparator: sham treatment. "For the control group, the ENT‐specialist will touch the tympanic membrane with an alligator ear forceps to simulate getting a paracentesis. In the same procedure, without having made a paracentesis, a ventilation tube is placed on the tympanic membrane and removed again afterwards."
Outcomes	Number of vertigo attacks lasting more than 20 minutes (assessed at 3 months and 24 months) Pure tone audiometry using a 4‐tone average at 500 Hz, 1000 Hz, 2000 Hz and 3000 Hz and a 3‐tone average at 125 Hz, 250 Hz and 500 Hz (assessed at 3 months) AAO‐HNS Functional Level Scale (assessed at 3 months) Hearing, tinnitus, unsteadiness and aural fullness (using a self‐evaluation questionnaire, assessed at 24 months) Number of participants discontinuing the trial (assessed at 24 months) Number of participants satisfied with treatment (assessed at 24 months) Speech audiometry (using speech discrimination, assessed at 3 months)
Starting date	20 September 2021 Estimated completion date: 1 January 2024
Contact information	Casper Grønlund Larsen: caslar@regionsjaelland.dk Bjarki Djurhuus: bjdd@regionsjaelland.dk
Notes	—

## Differences between protocol and review

Due to the paucity of data for most outcomes, we were unable to carry out a number of pre‐planned sensitivity analyses and subgroup analyses. We planned to use the Trustworthiness Screening Tool from Cochrane Pregnancy and Childbirth to identify studies for inclusion in the main analysis. However, as described in Selection of studies, we did not exclude studies from the main analysis on the basis of concerns whilst using this tool. We considered that the overall certainty of the review findings (all very low‐ or low‐certainty) would not be impacted by this decision. 
